# Exposure to COVID-19 is associated with increased altruism, particularly at the local level

**DOI:** 10.1038/s41598-021-97234-2

**Published:** 2021-09-23

**Authors:** Gianluca Grimalda, Nancy R. Buchan, Orgul D. Ozturk, Adriana C. Pinate, Giulia Urso, Marilynn B. Brewer

**Affiliations:** 1 Kiel Institute for the World Economy, Kiellinie 66, 24105 Kiel, Germany; 2 Sonoco Department of International Business, University of South Carolina, 1014 College Street, Columbia, SC 29205 USA; 3 Department of Economics, University of South Carolina, 1014 College Street, Columbia, SC 29205 USA; 4 Social Sciences, Gran Sasso Science Institute (GSSI), 67100 L’Aquila, Italy; 5 Department of Psychology, Ohio State University, 1835 Neil Avenue, Columbus, OH 43210 USA

**Keywords:** Psychology, Human behaviour, Social evolution

## Abstract

Theory posits that situations of existential threat will enhance prosociality in general and particularly toward others perceived as belonging to the same group as the individual (parochial altruism). Yet, the global character of the COVID-19 pandemic may blur boundaries between ingroups and outgroups and engage altruism at a broader level. In an online experiment, participants from the U.S. and Italy chose whether to allocate a monetary bonus to a charity active in COVID-19 relief efforts at the local, national, or international level. The purpose was to address two important questions about charitable giving in this context: first, what influences the propensity to give, and second, how is charitable giving distributed across different levels of collective welfare? We found that personal exposure to COVID-19 increased donations relative to those not exposed, even as levels of environmental exposure (numbers of cases locally) had no effect. With respect to targets of giving, we found that donors predominantly benefitted the local level; donations toward country and world levels were half as large. Social identity was found to influence charity choice in both countries, although an experimental manipulation of identity salience did not have any direct effect.

## Introduction

The persisting COVID-19 pandemic poses the most serious existential threat to contemporary societies since WWII, with wide-ranging consequences for cooperation and social cohesion within and between countries. Crises such as these heighten the tension between individual and collective interests^1^. On one hand, the stress and uncertainty of personal vulnerability make self-sacrifice more costly, particularly for those directly impacted. On the other hand, the shared threat enhances the salience of collective interdependence and the need to take collective interests into account. Human altruism is still a puzzle for both social sciences and evolutionary biology^2^, and its study in a situation of incumbent threat is valuable because it is precisely these situations that have characterized human societies for most of their evolutionary past^3^.

This paper addresses two inter-related questions. The first question concerns the extent to which people act altruistically rather than selfishly during the COVID-19 pandemic. The second question concerns the group toward which such altruism is directed.

We used the natural variation in exposure to the pandemic as a “quasi-experiment” to assess whether greater levels of exposure were associated with greater altruism, and whether such altruism had a parochial or cosmopolitan character. We used both a direct personal measure of exposure and an objective measure given by the county-level number of COVID-19 cases per inhabitant.

By parochialism we mean the tendency for individuals to direct altruistic acts toward others perceived as belonging to the same group as the individual. An “ingroup” is defined as a collectivity whose members experience some communality of fate, culture, or heritage which is associated with a sense of shared identity and attachment^4^. Research on parochial altruism typically focuses on the nation as the primary locus of ingroup attachment^5,6^. It is, however, unclear whether people would privilege even more local forms of attachment if given the opportunity. For this reason, in our experiment participants could donate money provided by the researchers to one charity active at the local, national, or global level in providing relief from COVID-19.

Theories of the evolutionary origin of prosociality conjecture that it is in situations of existential threat that individuals develop prosocial attitudes. It has been posited that under conditions of food scarcity, intergroup competition favored the development of both psychological dispositions and social norms oriented toward individuals sacrificing own interests to pursue group interests^3,7^. The reason is that groups that were able to achieve higher mutual self-sacrifice and cooperation were more likely to survive at times of scarcity. Contemporary research examining the aftereffects of exposure to natural disasters, such as earthquakes, tsunamis, or floods, however, reach conflicting results. While some studies suggest that community^8,9^ or country residents^10,11^ behave more prosocially in the short run following an experience with disaster, this effect may wane with time^12,13^. Other studies found null or negative effects^14,15^. It is then not clear whether exposure to natural hazards such as a pandemic will be associated with heightened prosociality among those affected^16–18^.

It is important to use multiple measures of exposure to the pandemic, because some people may experience COVID-19 directly—in person or through the diagnosis of someone close to them—while others may experience it only indirectly through its impact on the community and potential risk of exposure in the environment in which they live. Although personal exposure to near-fatal health threats has been associated with engaging in prosocial behaviour^19^ and the arousal of prosocial feelings^20^, heightened risk to the self has also been shown to reduce cooperation with others^19^. Moreover, little is known about the relative effects on altruism of personal exposure versus environmental-level exposure.

It has also been posited that prosociality triggered by existential threats will have a strongly parochial character. Situations of conflict were endemic to ancient societies, and outperforming another group in conflict ultimately required sacrificing individual resources, including one’s own life, for the ingroup at the expense of the outgroup^3,7,21^. A similar argument applies to exposure to diseases, which some see as a primary cause of intergroup competition^22^. Parasite-stress theory posits that greater exposure to diseases is associated with higher degree of ingroup favoritism and outgroup discrimination because of the threat that people from the outgroup will carry pathogens to which an individual is not immune^23^. Under conditions of threat from disease, group members are expected to increase preference for ingroup association and to have increased awareness of their shared fate and interdependence within the group^24^. Terror management theory^25^ also suggests that as life-threatening events occur, people will experience greater attachment to their ingroup because entrenching in traditional values reduces existential anxiety in a time of crisis and awareness of mortality^26^. Empirically, activating fear of death has been found to increase generosity in experimental games^27^ and contributions to ingroup charities^28^. Exposure to war has been associated with increased propensity to cooperate with one’s ingroup^29–32^, but not one’s outgroup, both at the individual and community level.

That prosociality during the COVID-19 pandemic is parochial is however not a foregone conclusion. While attempts by political leaders to depict COVID-19 as a “foreign virus” have been evident, when our study was conducted it was already clear that Asian countries where the virus had originated were no longer the locus of infection. Given that COVID-19 is a completely new strain of coronavirus, to which no group could have developed immunity in the past, contagion from one’s own group is, in principle, as likely as contagion from an outgroup.

Moreover, choices concerning prosociality during COVID-19, and many choices in general, often do not pit mutually exclusive ingroups and outgroups one against the other, but rather affect groups that are nested with each other and can be more or less inclusive^33^. One reason for this is that the public goods nature of combating a pandemic renders benefits non-excludable even to very distal others, although such benefits may be spatially concentrated. For instance, averting the pandemic in one country will also benefit other countries, although to a lesser degree. A second reason has to do with human construal of social identity. In contemporary societies, identities are often fluid and highly susceptible to being shaped by globalization^34^. It is precisely global shocks like COVID-19 that can trigger a stronger sense of “humanity as a whole”^35^, where “we are only as strong as the weakest among us”, as stated by UN Secretary General Guterres^36^.

The idea that humanity may be one’s ingroup has both theoretical foundations and empirical support^37,38^, and the hypothesis that globalization shapes an individual’s tendency to cooperate at different levels of inclusion has also been demonstrated^39,40^. Laboratory and field research on nested dilemma experiments suggest that individuals choose groups at higher levels of inclusiveness depending on relative social returns^41–43^, the availability of social information on others’ choices^42^, the level of saliency and attachment to the groups^43,44^, and the consequences of one’s action for other local groups^45^. Research with natural groups also shows varying propensity to cooperate with global others as opposed to local others^39^. The global nature of the COVID-19 pandemic may shift traditional loyalties and prompt individuals to include the whole of humanity as their ingroup. Thus, it is an open question whether individuals will become more or less parochial in their concern for others under conditions of global threat. We hypothesized that this decision would be influenced by the relative salience of social identities at different levels of inclusiveness, and our study included an experimental manipulation to test this hypothesis.

### Overview of present research

Our study consisted of two online experiments conducted in the U.S. (N = 932) and Italy (N = 723) during the first wave of the pandemic. Participants were given an unexpected monetary bonus ($5.00 in the U.S. and €4.00 in Italy) and asked whether they wished to donate some or all of the bonus and, if so, to which one of three charitable organizations providing aid to those affected by the COVID-19 pandemic. Participants had to first select one option between the four available—namely, (1) keep all the money for themselves; (2) Donate to a charity active at the state (in the U.S.) or region (in Italy), a level we label as “local”; (3) Donate to a national charity; (4) Donate to a global charity—and second, to decide how much of the bonus to donate to the charity (if the selected option was 2, 3, or 4) and how much to keep for themselves. We opted for this design because we wanted participants to express a clear-cut preference over the four options and to rule out the possibility that participants split their endowment evenly across the different charitable options. Any amount donated was matched by the researchers so that the contribution to the selected charity was doubled. Because of doubling, the donation decision had one basic property of a public goods dilemma: contributed funds resulted in increased benefits at the collective level (whether at the state, national or global levels) but a loss at the personal level for the individual donor. Yet, this choice lacked the strategic inter-dependence typical of social dilemmas, thus it most accurately reflects a measure of altruism rather than cooperation.

In order to assess the causal influence of construal of the ingroup on prosocial decision-making, our study included an experimental manipulation intended to “prompt” the participant to think about the pandemic crisis in terms of one of the three levels of inclusiveness. Empirically, priming identification has been shown to influence prosocial behaviors and increase cooperation in experimental social interactions^46^. Accordingly, we expected these prompts to increase identification with the corresponding level and, consequently, donation at that level.

## Results

Pre-registered hypotheses and a pre-analysis plan are available at the project repository: https://osf.io/jw46f/ (See Supplementary Note SN1).

We begin by presenting results on the main research questions presented above, analyzing first the relationship between exposure to the pandemic and willingness to donate in general. We then examine the amounts donated specifically to the three available charities at different levels of inclusiveness. To assess what factors, in addition to exposure, are related to which level of charity is chosen for donations, we analyze the role of social identity and of an attempted experimental manipulation of identity salience—which had been pre-registered as moderating factors of patterns of altruism. Finally, we report an exploratory analysis of what charity characteristics are most closely associated with the choice of charity for donations.

### Predictors of choosing to donate

In both countries, a majority of survey respondents were willing to forego some or all of their bonus money to contribute to collective welfare. In the U.S., 63% of survey participants chose to donate at least some of their bonus to a charity; in Italy, 77% of participants made a donation. For those who chose to donate, the average donation amount in the U.S. was $2.75 (0.55 of $5 bonus fund) and in Italy €2.48 (0.63 of €4 bonus fund). Overall, 40% of the bonus money was donated to the charities.

### Personal exposure, and not county-level exposure, predicted giving

We used a hurdle model to assess simultaneously the effects of COVID-19 exposure on both the probability of choosing to donate to a charity (*P*) and conditional donations (*CD*), that is, the amount donated conditional on being a donor (see "Methods"). The model included a set of demographic variables, the participant’s political orientation and area of residence, and the exogenously assigned prompt treatment (see Supplementary Information: Supplementary Table 1 for descriptive statistics of the variables). We used as our measure of environmental exposure to disease the county-level count of cases per 100,000 inhabitants in the county where the participant resided (see "Methods"). This environmental exposure measure proved to have no significant effect in the U.S. on either *P* (Average Marginal Effect—AME henceforth = 0.001; *p* = 0.99) or *CD* (AME = 0.00; *p* = 0.74). In Italy, it had no significant effect on *P* (AME = 0.001; *p* = 0.59) and was at the margin of statistical significance for *CD* (AME = 0.003; *p* = 0.098) (see Table 1, columns 1–2 and 5–6).

**Table 1:** Econometric analysis of probability of being a donor (*P*) and of conditional donation (*CD*).

DEP VAR	United States				Italy			
	Model 1		Model 2		Model 1		Model 2	
	*P*	*CD*	*P*	*CD*	*P*	*CD*	*P*	*CD*
	(1)	(2)	(3)	(4)	(5)	(6)	(7)	(8)
Age	0.003**	0.003**	0.003**	0.004**	0.002	0.001	0.002	0.001
	[0.001]	[0.001]	[0.001]	[0.001]	[0.001]	[0.002]	[0.001]	[0.002]
Female	0.101***	0.104***	0.097***	0.100**	0.055*	0.049	0.053*	0.046
	[0.023]	[0.031]	[0.023]	[0.031]	[0.026]	[0.031]	[0.026]	[0.031]
Conservative scale	− 0.051***	− 0.058***	− 0.049***	− 0.055***	− 0.092***	− 0.088***	− 0.090***	− 0.086***
	[0.010]	[0.013]	[0.010]	[0.013]	[0.014]	[0.016]	[0.014]	[0.016]
Income	0.019***	0.015+	0.018**	0.013+	0.006	0.007	0.006	0.007
	[0.006]	[0.008]	[0.006]	[0.008]	[0.007]	[0.008]	[0.007]	[0.008]
County-level COVID Exposure	0.001	0.00	0.001	0.00	0.001	0.003+	0.001	0.003
	[0.002]	[0.003]	[0.002]	[0.003]	[0.002]	[0.002]	[0.002]	[0.002]
Personal COVID Exposure			0.058*	0.077*			0.045+	0.054+
			[0.024]	[0.033]			[0.027]	[0.032]
LR chi2	88.66	48.62	95.04	53.96	83.81	41.23	87.07	44.05
Observations	932	932	932	932	723	723	723	723

Estimates of average marginal effects (AME) from two-part hurdle models are reported. The dependent variable is the share of bonus donated to a charity, without identifying which charity had been chosen. *CD* is the amount donated conditional on being a donor. The first column in each model reports the marginal effects from a Probit model to estimate *P*. The second column in each model reports the marginal effects for *CD*. In addition to the covariates reported above, all models also include controls for education level, indicator for size of respondent’s location, income, whether the individual reported an income loss because of COVID-19, priming (state/country/world), macro-region dummies (South/North for Italy and South/Midwest/Northeast/West for the U.S.), and an indicator identifying individuals who were either born abroad or whose parents were born abroad. The full regression output is reported in the Supplementary Table 2a. Variables are defined in Supplementary Table 1. Standard errors are in brackets.

*** = *p* < 0.001, ** = *p* < 0.01, * = *p* < 0.05, +  = *p* < 0.10.

**Figure 1:**
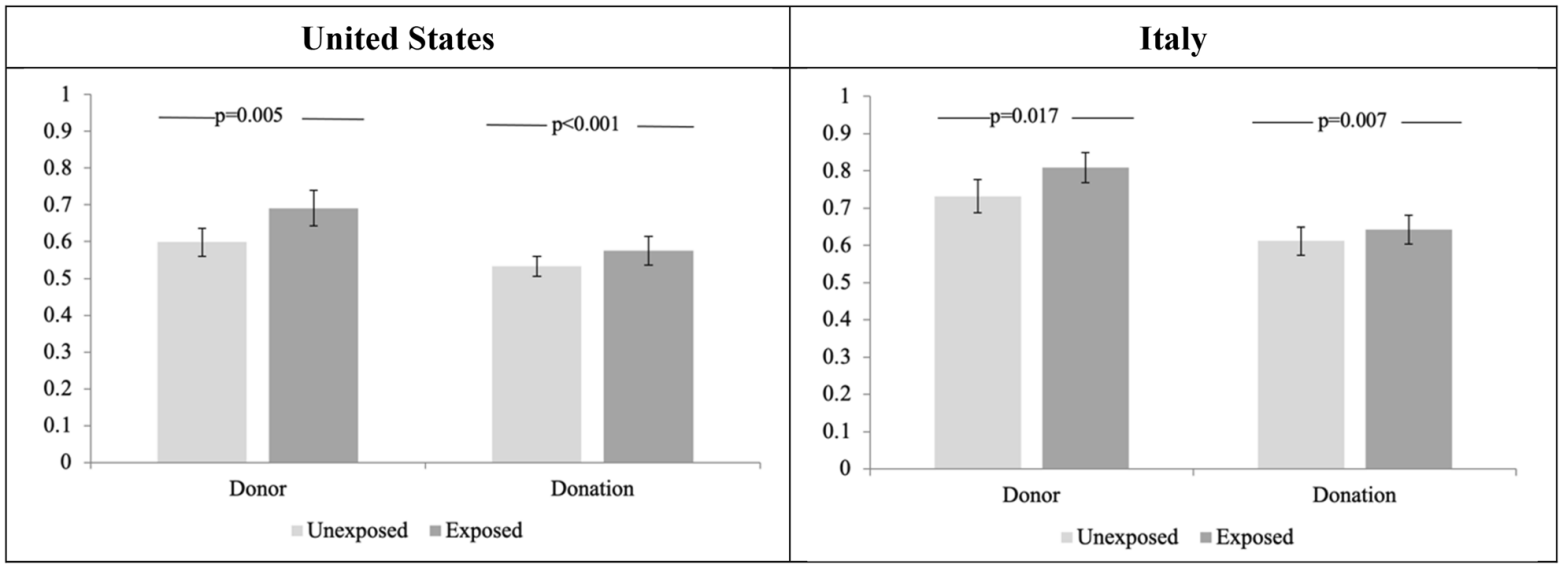
Impact of personal exposure to COVID-19 on frequency of donations and amount donated. **‘**Donor’ is a dichotomous variable taking value of 1 if a participant donated a positive sum to a charity, and 0 otherwise. ‘Donation’ is the amount donated, as a share of the bonus available for donation. The two panels report the means of the two variables, broken down by participants personally unexposed and exposed to COVID-19 in U.S. and Italy. Participants were identified as “Exposed” if they, their family members, or their acquaintances, had been diagnosed with or had died from COVID-19. Unexposed participants were all others. The whiskers denote 95% confidence intervals obtained from bootstrapped errors with 10,000 repetitions. The *p*-values report significance levels of a Fisher exact test (for ‘Donor’) and of a Mann–Whitney Wilcoxon test (for ‘Donation’) that the observations for Exposed and Unexposed individuals come from the same distribution.

We conjectured that the lack of significant effects may have been due to county-level data providing only a coarse, though the most disaggregated available, measure of exposure. It is plausible that only when an individual or their close acquaintances are personally afflicted by the disease does the perception of the threat of the disease become psychologically compelling. In fact, county-level exposure and personal exposure are uncorrelated (Cohen’s d–d henceforth- = 0.07; CI = [− 0.04; 0.17]; Pearson’s *r* = 0.03). We therefore added to our pre-analysis plan a dummy variable for participants’ self-reported personal exposure. Participants were identified as “exposed” if they, their family members, or their acquaintances, had been diagnosed with, or had died from COVID-19 (see "Methods" and Supplementary Table 1). This variable was positively and significantly associated with *P* both in the U.S. (*p* = 0.020; d = 0.19; CI = [0.06; 0.33]) and—marginally—in Italy (*p* = 0.093; d = 0.18; CI = [0.04; 0.33]), as well as with *CD* both in the U.S. (*p* = 0.016; d = 0.22; CI = [0.08; 0.36]) and—marginally in Italy (*p* = 0.092; d = 0.20; CI = [0.05; 0.35]; see Table 1, columns 3–4 and 7–8 and Fig. 1 for means). When the data for the two countries were combined into one analysis, the COVID-19 personal exposure effect was significant for both *P* (*p* = 0.005; d = 0.22; CI = [0.12; 0.32]) and *CD* (*p* = 0.002; d = 0.25; CI = [0.15; 0.35]) and there was no significant difference between countries in the size of this effect (AME =  − 0.012; *p* = 0.82; for *P*, and AME =  − 0.011; *p* = 0.73; for *CD*) (see Supplementary Table 2c).

**Figure 2:**
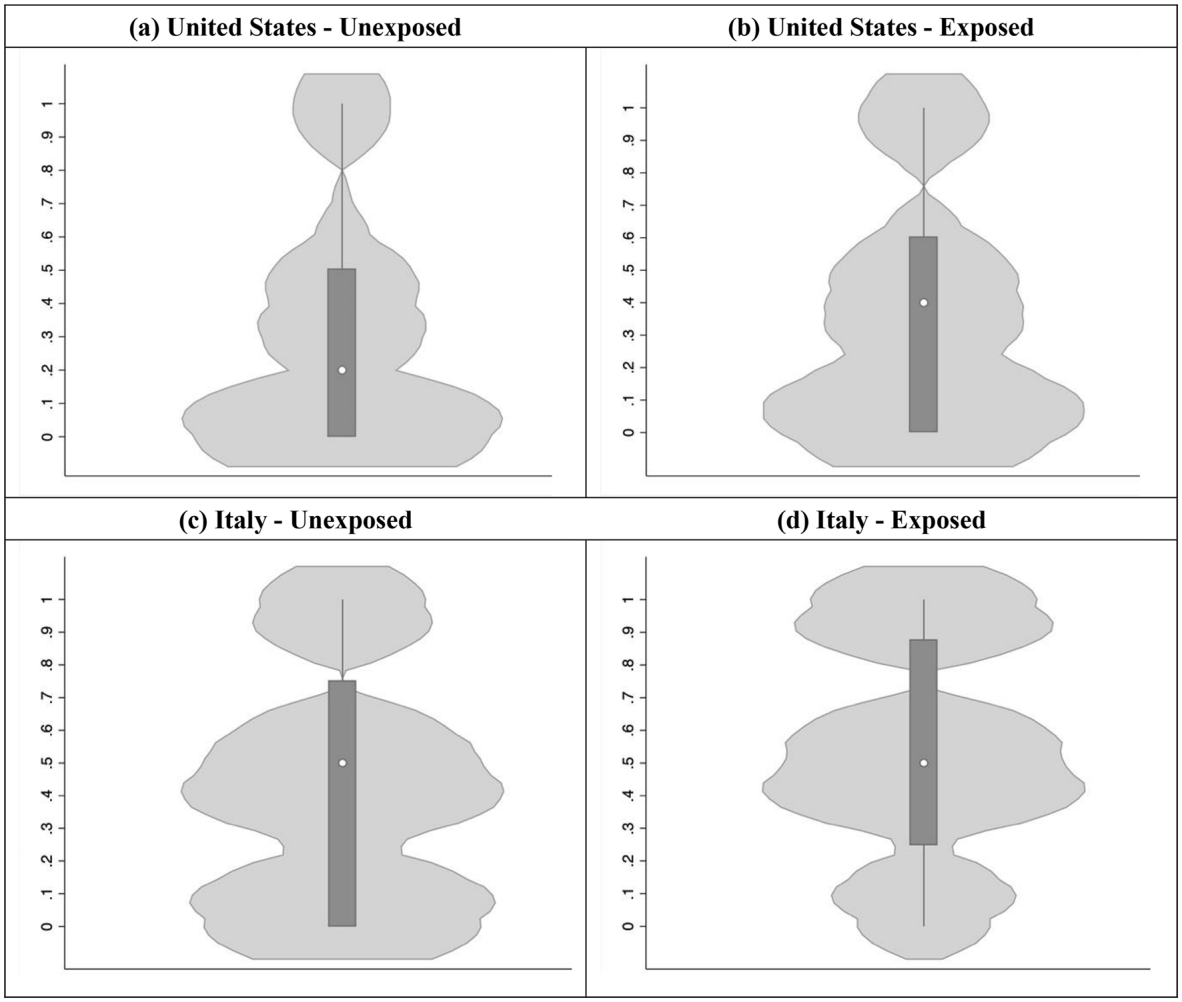
Distribution of amount donated by country and exposure to COVID-19. Distribution plots include a white point indicating the median of the distribution, a box indicating the interquartile range (from 25th percentile up to 75%) percentile, and spikes extending to the upper- and lower-adjacent values. Overlaid with this box plot is the density of the distribution, estimated by k-density.

Figure 2 provides a graphic representation of the distribution of contribution decisions for personally exposed and non-exposed respondents. It is of interest to note that willingness to donate 100% of the bonus money was greater for the personally-exposed participants, and this was particularly the case in Italy. For respondents in the U.S., having personal contact with the illness increased both the probability of deciding to donate by 9% and the average donation by 9.2% of the bonus. The marginal effects were similar in Italy, with personal exposure increasing the probability of donating by 7.5% and the amount donated by 5.8% of the bonus. Thus, a significant effect of personal exposure to COVID-19 was replicated across the two countries, affecting both the propensity to donate and the amount given.

### Distribution of charity donations and effects of exposure

**Figure 3:**
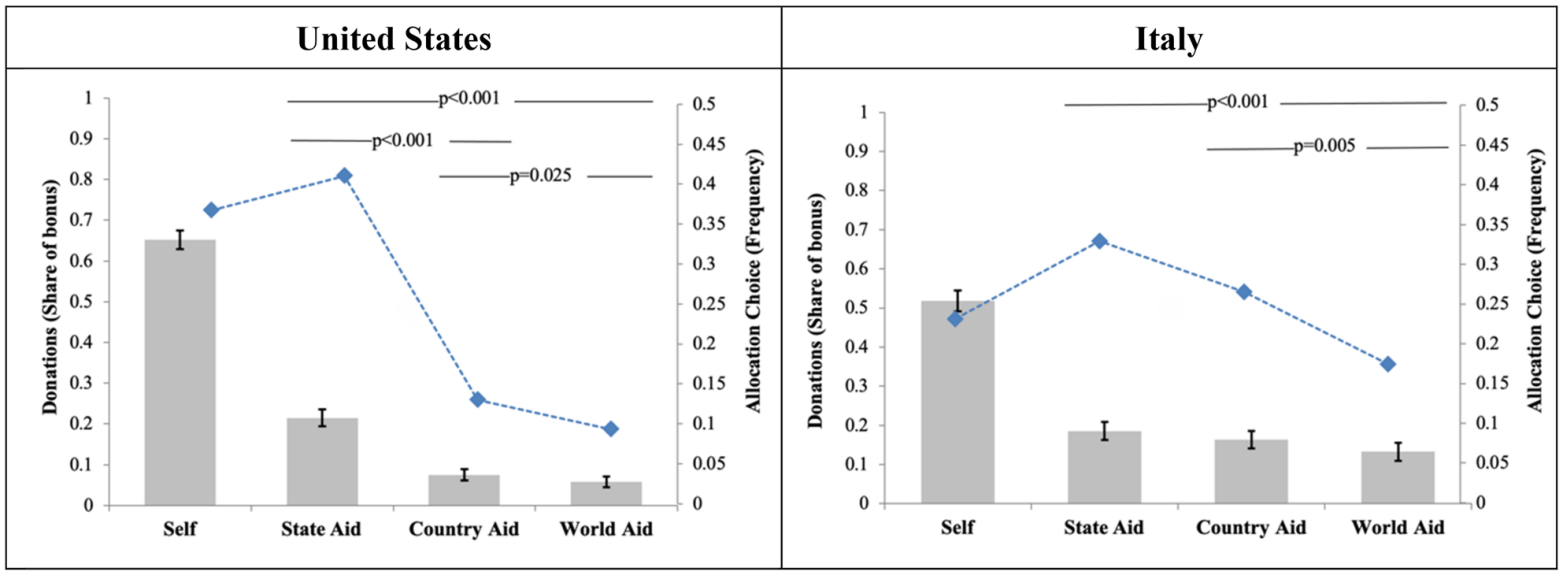
Patterns of bonus allocation. The frequency with which each charity is selected (blue diamonds, scale on the right-hand y-axis) and the mean levels of aggregate donations (*AD*) to each charity and the amount kept for self (bars, scale on left-hand y-axis) are plotted. Whiskers indicate the confidence intervals for mean *AD*, obtained from bootstrapped standard errors with 10,000 repetitions. The *p*-values report significance levels of t-tests on the null hypothesis of equality of pairs of coefficients estimated in Supplementary Table 4, columns 1–2. Significance levels for pairwise tests between allocation to self and contribution to charities, which are in all cases significant at *p* < 0.001, are not plotted.

In both countries, the modal option for donations was to donate to the charity at the most local level—namely, the participant’s state of residence in the U.S. and region of residence in Italy. As shown in Fig. 3, in the U.S. 41.0% donated to the state charity and in Italy 32.9% donated to the regional charity. The national charity was more frequently selected in Italy (26.6% of the sample) than in the U.S. (13.0%), and the same pattern occurred for the international charity, which was selected by 17.4% of participants in Italy and 9.33% in the U.S.. We call ‘Aggregate Donations’ (*AD* henceforth) the overall amount of money allocated to each of the four options (i.e. self and the three charities). *AD* offers a comprehensive measure of the money allocated to each charity, as it combines both the extensive margin (which charity is chosen) and the intensive margin (how much money is donated conditional on choosing a certain charity).

In the U.S., 65.2% of the bonus money available was kept for oneself, 21.5% went to the state-level charity, while 7.5% and 5.8% of *AD* were allocated to the national and international charity, respectively. Using a repeated-measures Tobit model having the same covariates as the model used previously, we find that *AD* allocated to the state-level charity were significantly higher than both country-level *AD* (*p* < 0.001; d = 0.50; CI = [0.41; 0.60]) and world-level *AD* (*p* < 0.001; d = 0.59; CI = [0.49; 0.68]). Country-level *AD* were also significantly higher than world-level *AD*, but with much lower effect size (*p* = 0.025; d = 0.08; CI = [− 0.01; 0.17]) (Supplementary Table 4).

In Italy, 51.8% of bonus money was kept, while 18.6% went to the regional charity. 16.3% and 13.2% of Italian participants allocated their *AD* to national and international charities, respectively. *AD* were more evenly distributed, and effect sizes were smaller, in Italy than in the U.S.. *AD* to the regional charity were not significantly different, at conventional levels, than *AD* to national charities (*p* = 0.080; d = 0.07; CI = [− 0.03; 0.17]), but *AD* to the world charity were significantly lower than *AD* to the regional charity (*p* < 0.001; d = 0.17; CI = [0.07; 0.28]) and to the national charity (*p* = 0.005; d = 0.10; CI = [− 0.002; 0.20]). *AD* allocated to state-level charities in the U.S. were significantly higher than *AD* allocated to regional charities in Italy (*p* = 0.018; d = 0.30; CI = [− 0.01; 0.19]). Italian participants donated significantly more to national charities (*p* < 0.001; d = 0.34; CI = [0.23; 0.43]) and to international charities (*p* < 0.001; d = 0.29; CI = [0.20; 0.39]) than U.S. participants with small to medium effect size (Supplementary Table 4).

As for our research question of whether exposure to COVID-19 was associated with parochial or cosmopolitan giving, county-level exposure to COVID-19 did not significantly predict *AD* at any level of choice in multivariate Tobit and Probit models (see "Methods" and Supplementary Table S5a–d). Personal exposure was at the margins of statistical significance for local charity giving in the U.S. (*p* = 0.086; Table 2, column 1) with a very small effect size (d = 0.15; CI = [0.01; 0.28]), but no significant effect in Italy (*p* = 0.31; d = 0.08; CI = [− 0.06; 0.23]). Pooling the two countries returned a statistically significant effect for personal exposure on local giving (*p* = 0.025, Table S5b, column 1), albeit with a very small effect size (d = 0.11; CI = [0.005; 0.21]). Personal exposure had no predictive power on national giving in any specification being used (Supplementary Table S5a–d) with negligible effect sizes. Personal exposure was at the margins of statistical significance in Italy in predicting global giving (*p* = 0.073, Table 2, column 9) with a small effect size (d = 0.21; CI = [0.06; 0.35]), but was insignificant in the U.S. (*p* = 0.54; d = 0.04; CI = [− 0.09; 0.17]). The effect was at the margins of significance pooling the two countries (*p* = 0.058; d = 0.16; CI = [0.06; 0.26]).

**Table 2:** Econometric analysis of Aggregate Donations (*AD*).

DEP VAR: AD	United States						Italy					
	Model 1			Model 2			Model 1			Model 2		
	State	Country	World	State	Country	World	Region	Country	World	Region	Country	World
	(1)	(2)	(3)	(4)	(5)	(6)	(7)	(8)	(9)	(10)	(11)	(12)
Age	0.009***	− 0.011*	0.00	0.008***	− 0.011*	0.002	0.009**	− 0.011**	0.002	0.007*	− 0.010*	0.005
	[0.002]	[0.005]	[0.006]	[0.002]	[0.005]	[0.006]	[0.003]	[0.004]	[0.006]	[0.003]	[0.004]	[0.006]
Female	0.131**	0.124	0.086	0.138**	0.117	0.07	0.144*	0.06	− 0.127	0.165*	0.043	− 0.142
	[0.049]	[0.108]	[0.135]	[0.048]	[0.107]	[0.133]	[0.067]	[0.081]	[0.123]	[0.067]	[0.081]	[0.121]
Conservative scale	− 0.032	− 0.095*	− 0.183**	− 0.045+	− 0.121*	− 0.126+	0.084*	− 0.051	− 0.627***	0.044	− 0.062	− 0.511***
	[0.021]	[0.047]	[0.063]	[0.023]	[0.054]	[0.069]	[0.036]	[0.046]	[0.088]	[0.037]	[0.048]	[0.084]
Personal COVID Exposure	0.087+	0.076	0.085	0.065	0.069	0.051	0.069	− 0.089	0.227+	0.056	− 0.059	0.186
	[0.051]	[0.111]	[0.139]	[0.050]	[0.112]	[0.139]	[0.068]	[0.083]	[0.127]	[0.067]	[0.083]	[0.125]
Priming State/Region	− 0.022	− 0.071	− 0.134	− 0.033	− 0.078	− 0.113	0.169+	− 0.051	− 0.069	0.198*	− 0.098	− 0.099
	[0.068]	[0.151]	[0.196]	[0.067]	[0.150]	[0.195]	[0.094]	[0.110]	[0.179]	[0.092]	[0.109]	[0.174]
Priming Country	− 0.091	− 0.048	− 0.083	− 0.09	− 0.064	− 0.045	0.118	− 0.087	0.084	0.128	− 0.096	0.039
	[0.069]	[0.149]	[0.194]	[0.067]	[0.148]	[0.192]	[0.096]	[0.113]	[0.176]	[0.094]	[0.111]	[0.171]
Priming World	− 0.06	− 0.121	0.228	− 0.067	− 0.12	0.256	0.125	− 0.268*	0.372*	0.133	− 0.287*	0.331*
	[0.067]	[0.147]	[0.180]	[0.066]	[0.146]	[0.179]	[0.094]	[0.117]	[0.168]	[0.092]	[0.115]	[0.163]
Local Social Identity				0.214***	− 0.208**	− 0.270**				0.298***	− 0.271***	− 0.132
				[0.035]	[0.080]	[0.102]				[0.055]	[0.069]	[0.100]
National Social Identity				− 0.021	0.202*	− 0.037				− 0.119*	0.315***	− 0.218*
				[0.038]	[0.088]	[0.108]				[0.055]	[0.071]	[0.101]
Global Social Identity				− 0.053	0.093	0.292**				− 0.095*	− 0.049	0.489***
				[0.033]	[0.069]	[0.089]				[0.048]	[0.060]	[0.102]
LR chi2	100.34			174.58			178.66			270.91		
Observations	932			932			723			723		

We fit multivariate Tobit models to estimate *AD* for each of the three charities. *AD* is the overall amount of donations to each charity, combining both the extensive margin (which charity is chosen) and the intensive margin (conditional donations to each charity). In addition to the covariates reported above, all models also include controls for education level, indicator for respondent’s location size, income, whether the individual reported an income loss because of COVID-19, macro-region dummies (South/North for Italy and South/Midwest/Northeast/West for the U.S.), an indicator identifying individuals who were either born abroad or whose parents were born abroad, and county-level exposure to COVID-19. The full regression output is reported in the Supplementary Table 5a. Variables are defined in Supplementary Table 1. Standard errors are in brackets.

***p < 0.001, **p < 0.01, *p < 0.05, + *p* < 0.10.

### People choosing the world charity donated significantly more than those choosing other charities

**Figure 4:**
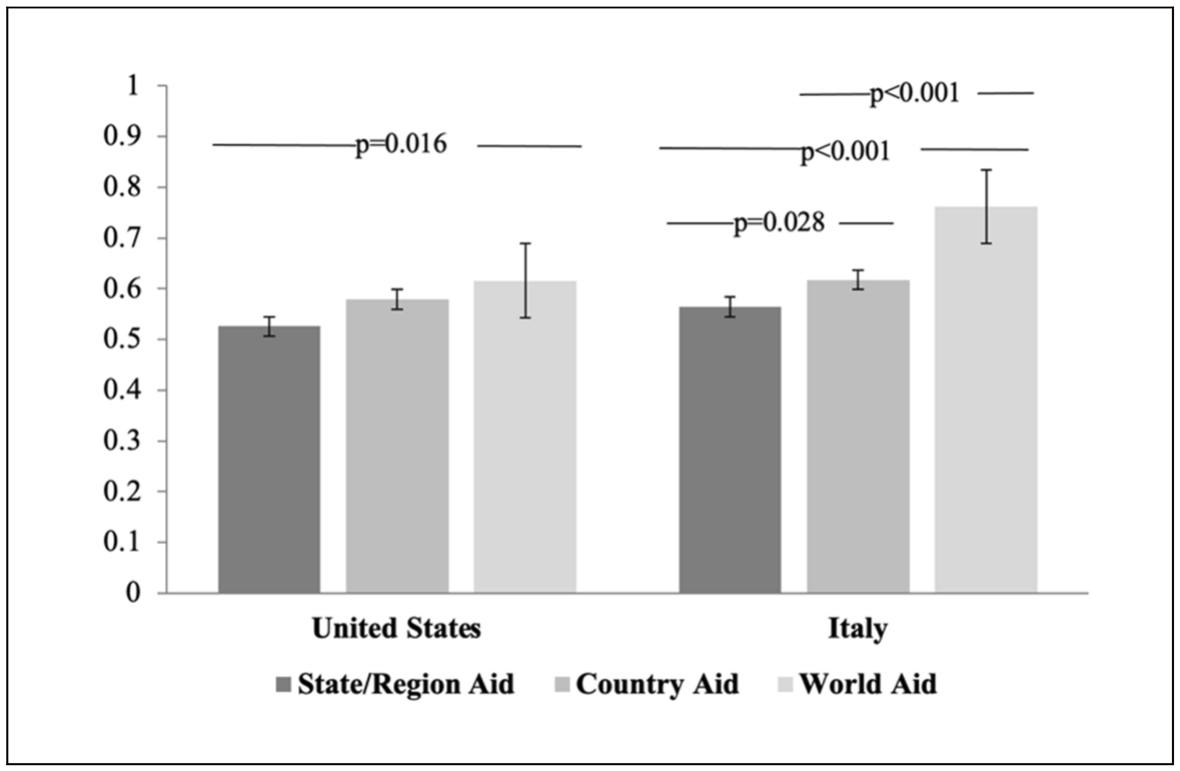
Conditional donations. The shares of bonus donated to a charity, conditional on donating, are plotted. The *p*-values report significance levels of Mann–Whitney-Wilcoxon tests on the null hypothesis that observations come from the same distribution. Whiskers indicate the confidence intervals for mean *CD*, obtained from bootstrapped standard errors with 10,000 repetitions.

We analyzed *CD*—the amount donated conditional on being a donor—with respect to the distribution of allocations to the three different charities. *CD* to the world charity were the highest among the three in both countries, followed by *CD* to the national charity, and *CD* to the state/regional charity (Fig. 4). *CD* to the international charity were significantly higher than *CD* to the state charity in the U.S. (*p* = 0.007; d = 0.30; CI = [0.06; 0.54]), and significantly higher than either *CD* to regional charities (*p* < 0.001; d = 0.72; CI = [0.48; 0.95]) or national charities (*p* < 0.001; d = 0.53; CI = [0.29; 0.77]) in Italy (Supplementary Table 2b). In other words, participants who selected the world charity gave more than participants who selected the state or regional charities. Therefore, the finding that *AD* were highest for the state/regional charity was driven more by which charity was chosen by participants, rather than by how much was given.

### Prompting had limited effects on where donations were directed

As described in the "Methods" section, each participant was randomly assigned to a different framing condition aiming to prompt individuals to portray COVID-19 as a problem for (a) the state of residence (in the U.S.) or region of residence (in Italy) (*Local Prompt* henceforth), (b) the country (*National Prompt*), or (c) the world (*World Prompt*). In the Control condition, no geographical connotation was provided.

After ascertaining the exogeneity of the prompt to the main demographic characteristics of the samples (Supplementary Table 6), we used a multivariate Tobit model to analyze the effect of the three prompts on aggregate donations at the local, national or world level. This model enables us to capture the interdependent nature of the charity choice for donation (see "Methods"). We found that none of the prompts increased donations significantly in the U.S. in comparison to the Control condition. This was the case for each of the three levels of donation, using the same covariates as in our previous models (Table 2, columns 1–3). In Italy (see Table 2, columns 7–9), the *World Prompt* consistently had a significant effect in increasing donations to the world charity (*p* = 0.027) with very small effect size (d = 0.16; CI = [0.04; 0.36]), while also having a negative effect on national donations (*p* = 0.022; d = 0.21 CI = [0.002; 0.42]). The *National Prompt* had no effect on national donations (*p* = 0.44; d =  − 0.08; CI = [− 0.29; 0.13]), while the *Local Prompt* was at the margins of significance at conventional levels and had small effect size in increasing contributions to the local charity (*p* = 0.073; d = 0.18; CI = [− 0.03; 0.38]).

Overall, then, the prompt manipulation proved to have little influence on donation decisions and apparently was not powerful enough to override participants’ prior perspective on the scope of the pandemic crisis. Nor did it affect the predicted mediator of social identification at the different levels (see Supplementary Table 7, and Supplementary Note SN2).

### Social identity predicted donation choice

In experimental research on social dilemmas, the strength of social identification with an ingroup increases intragroup cooperation^47,48^. Social identity has been found to be a relevant factor to explain cooperation in a nested social dilemma game, particularly at the global level^40^. As laid out in our pre-registration plan, we conjectured that the same would be the case for donation behavior and that social identification with local, national, and global groups would relate to giving to charities at the different levels. Thus, further analyses were conducted to look at the effects of social identification itself, independent of the prompts.

**Figure 5:**
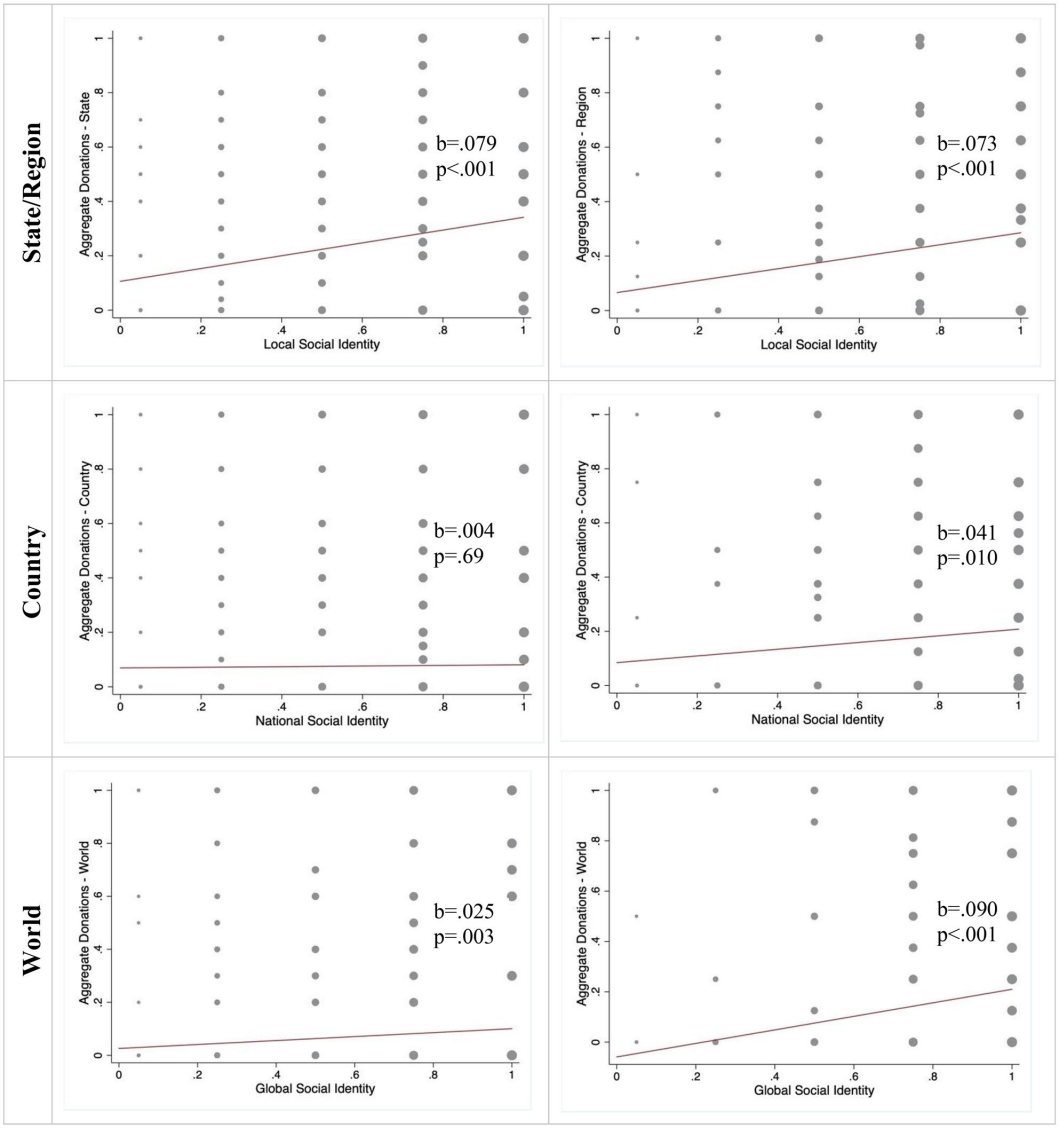
Relationship between social identity and corresponding level of aggregate donations. We plot the relationship between the three social identity scores (x-axis) and aggregate donations (y-axis). We grouped social identity scores on five, equally sized, bins. The size of the dots is determined by observed frequencies. The red lines are the OLS-best-fit interpolating lines. *b* is the slope of the interpolating line and *p* is the significance level of a t-test that the slope is equal to zero.

Figure 5 displays the relationship between strength of social identity at each level and aggregate donations at the corresponding level for U.S. and Italy. We employed a Tobit multivariate model to predict *AD* from social identity, using the same set of covariates used in previous models. Our expectations were confirmed in that social identity at each level was a significant predictor of donation at that level. This was the case for local giving both in the U.S. (*p* < 0.001; d = 0.28; CI = [0.15; 0.41]) and Italy (*p* < 0.001; d = 0.27; CI = [0.11; 0.43]); national giving in the U.S. (*p* = 0.021; d = 0.08; CI = [− 0.05; 0.20]) and Italy (*p* < 0.001; d = 0.24; CI = [0.06; 0.41]); global identity in the U.S. (*p* = 0.001; d = 0.21; CI = [0.08; 0.34]) and Italy (*p* < 0.001; d = 0.42; CI = [0.32; 0.52]) (Table 2, columns 4–6 and 10–12). Moreover, we found that there were no significant differences in the effects of social identity in the two countries in a pooled model (Supplementary Table 5b), corroborating the robustness of this result. Similar results were obtained analyzing the effect of social identity on the probability of choosing one of the three charities (Supplementary Tables 5c–d). The effect of social identity was also robust to the inclusion of additional possible explanatory factors, such as trust in other people (see Supplementary Tables 5a–b and Supplementary Note SN3).

### Donations were motivated by concern for others’ needs and charity efficacy

While social identity offers a general explanation for altruistic behavior that could span several contexts, the charity choice decision made by our participants may also have been influenced by more specific factors connected with their perceptions about charities at each level. First, a participant may have been motivated to give to a charity expecting to be on the receiving end from that charity’s activity in the future. This may explain the larger share of overall giving to local charities. In other words, people may expect that their Per Capita Return—i.e. the level of personal benefit from donations—would be higher for the local charities than the national and the global charity. Several laboratory experiments confirm that individuals are indeed sensitive to the Per Capita Return when giving to a public good^41^—even when the choice of giving runs against their self-interest, as in our experiment.

Alternatively, according to generalized bounded reciprocity theory, people are motivated to cooperate by the expectation that other people within the group will also cooperate^49^. If this motivation was active in our experiment, we would then expect people to donate at the level where they most expect others to donate. Other possible accounts concern the perceived capacity of a certain charity to achieve its goals, and its efficiency in meeting goals without wasting money^50^. Finally, people may be motivated by a purely altruistic desire to help people most in need because of the effects of COVID-19. Perceived need has been found to be a strong motivator of prosocial behavior^51^.

Preliminary data relevant to these questions about motives for donation were obtained from analyses of responses to an open-ended question at the end of the survey questionnaire in the U.S. survey. The question asked participants to give a short answer about why they made the decision to donate or not. No responses provided by participants made explicit reference to expectations that any of the charities would benefit themselves. Among those who chose to donate, 56% mentioned others’ need or wanting to help others as their reason for giving. In addition, a small percentage (4%) mentioned perceived effectiveness as their reason for choosing a particular charity and most of these referred to the state level.

To pursue this more systematically, the Italian survey included a set of structured questions regarding specific characteristics of charities at the regional, country, or world level that may have affected giving behavior. (This analysis was not part of our pre-analysis plan, so it should be considered as supplementary to our hypothesis-testing results). We had one item for each of the possible factors mentioned above: perception of (a) Per Capita Return, (b) bounded generalized reciprocity, (c) charity’s effectiveness and (d) efficiency, and (e) awareness of need (see Supplementary Table 1 for item wording and Supplementary Note SN4 for details on the analysis).

We applied the same multivariate Tobit model used in the previous section to explain *AD*, adding the five items jointly to the regression (see Supplementary Table 8). We did not find support for the idea that expecting individual benefit predicted donations at any level, whether the regional charity (b = 0.13; *p* = 0.37), the national charity (b =  − 0.08; *p* = 0.39), or the international charity (b =  − 0.08; *p* = 0.78) where b is the marginal effect of the independent variable on the latent index of willingness to donate estimated in Supplementary Table 8. Likewise, the expectation that people were motivated by their expectations of what others would donate did not receive support for any level of donation (b = 0.05, *p* = 0.76 for the regional level; b =  − 0.082, *p* = 0.053 for the national level; b = 0.02, *p* = 0.66 for the world level). Support was found for the influence of the other three factors. The perceived characteristic having the highest weight was the perception of a charity’s effectiveness in pursuing its goal of providing relief from COVID-19: participants who rated a specific level of charity as most effective gave significantly more at the corresponding level, particularly at the world level (b = 0.40, *p* = 0.008 for regional level; b = 0.52, *p* < 0.001 for national level; b = 0.79, *p* < 0.001 for world level). The perception of charity efficiency was also significantly related to donations at the regional level (b = 0.47; *p* = 0.001), country level (b = 0.55; *p* = 0.041), and world level (b = 0.55; *p* = 0.001). Finally, the perception of people’s needs was significantly related to donations at the respective levels, particularly at the regional and the world levels (b = 0.73, *p* = 0.001 for regional aid; b = 0.19, *p* = 0.31 for national aid; b = 0.75, *p* < 0.001 for international aid). Overall, it seems that participants had truly altruistic concerns in benefitting those charities better capable of providing relief and helping those in greater need, while assessments of which charity may benefit themselves in the future had a limited role.

All of the above results hold controlling for several demographic variables, political orientation, and other attitudinal characteristics. Their effects are described in Supplementary Note SN3^52^. We also examined the possibility of experimenter demand effects associated with our framing manipulation but found little evidence that participants guessed experimenter intent or that such guesses influenced their choice of charity (Supplementary Note SN5).

## Discussion

Giving to a charity is the quintessential altruistic action, as individuals give up resources to benefit unknown others. It has been posited that such altruism rests on psychological dispositions to help others in conditions of threat to the community and resource scarcity, as favored by multi-level selection forces in situations of inter-group conflict^3,7^. Our finding that people donated a significant amount of resources in a situation of existential threat like the COVID-19 pandemic is consistent with such theories. In our case, donations were specifically directed to relief efforts related to the pandemic. It is an open question whether these altruistic tendencies would generalize to other forms of prosocial behavior.

Our evidence that direct personal exposure to the pandemic is associated with greater giving is also consistent with this theory and contributes to our understanding of specific factors influencing prosociality in times of crisis. A complementary explanation is that when people are personally exposed to the pandemic, feelings of empathy for those closest to them extend to unknown others^19^, albeit primarily in a parochial manner. No evidence of a significant effect of environmental exposure to the disease on altruistic behavior is found when we measure the actual number of cases in the participant’s area. This suggests that the subjective psychological construal of the crisis is somehow independent from the “objective” threat faced by people in their area of residence^53^, and that direct personal exposure acts as a factor to activate prosocial behavior. It was only when the pandemic hit closest to home—affecting those known to the respondent—that exposure increased willingness to contribute to a collective good. It is possible that personal exposure makes the reality of the pandemic more certain and the need for charity more obvious. It is also of note that personal exposure was weakly associated with increased local giving in the U.S. and increased global giving in Italy.

Several patterns in our study are observed in both the United States and Italy, lending robustness to our results. In particular, the willingness to benefit most the group at the lowest levels of inclusiveness, the tendency for people more personally exposed to COVID-19 to donate more, and the moderating effect exerted by social identity on charity choice are found in both countries at comparable levels of intensity. Nonetheless, contributions to more inclusive groups—country and world—were also substantial and larger in Italy than the U.S.—suggesting that inclusiveness may be affected by contextual factors such as the uneven regional distribution of the threat in question, the time of the outbreak, and the nature of governmental response to the pandemic.

A speculative conjecture is that the circumstances surrounding responses to the COVID-19 pandemic in the U.S. made the state and county levels of government largely responsible for policies implemented to curb the spread of the disease. Consequently, interdependence at the state level was made particularly salient and the state became the social unit most likely to elicit expectations of mutual aid. In Italy, on the other hand, the response to the pandemic was largely directed from the national level of government, making national identification more salient.

Another pattern that is consistent across both countries is the finding that those who donated to world charities donated more than others. These results are consistent with previous research showing that people having cosmopolitan attitudes are overall more generous than others^38,40,54^. It is an open question whether the levels of giving observed in times of the pandemic will be maintained over time. It has been argued that wars have long-lasting positive effects on people’s trust^55^. It has also been shown that exposure to shocking events, such as wars, pandemics, and natural disasters, have an impact on social norms that can persist for decades and is transmitted to subsequent generations, especially when such shocks impact individuals at an early age^55–57^. Clearly, more research is needed on this point.

Although we cannot draw direct policy implications from the present study, we believe that the evidence provided may inform the policy debate in several directions. While our study signals the existence of a substantial portion of people willing to contribute to the common good, the largest proportion of resources are still kept for the self or the local community. Many global leaders have lamented the current failure of multi-lateral governance in addressing the spread of COVID-19, let alone facing up to other global challenges affecting our planet. Our findings suggest that whatever global effort is constructed to address the current and other ongoing world-wide crises, it will have to consider the markedly parochial character of prosociality observed in our study.

## Methods

Experiment protocol, experiment instructions and questionnaire have been deposited at the project depository at the Open Science Foundation: https://osf.io/jw46f/.

### Ethical approval

Our research plan was approved by the Institutional Review Board for Human Subjects at the University of South Carolina (Pro00099715) for the U.S., and by the Reggio Emilia Behavioral and Experimental Laboratory for Italy. All research was performed in accordance with relevant guidelines/regulations and in accordance with the Declaration of Helsinki. Participation in the research was voluntary and informed consent was obtained from all participants.

### Period of fieldwork and recruitment methods

The survey was conducted during the week between 13 and 20th of May 2020 in the United States. Respondents (N = 932) were recruited from the Prolific worker pool, screened to include only U.S. citizens or permanent residents and by the use of quota sampling to achieve equal participation across the four Center for Disease Control (CDC) regions of the U.S. and two age groups ([18–30], [over 30]).

For purposes of replication and generalization of results, a replication of the survey was conducted in Italy between the 11th and 23rd of June 2020. At this time, the situation in the U.S. and Italy was quite similar in terms of registered deaths per population (see Supplementary Fig. S1)^58^. Much of the U.S. was still dealing with the pandemic as an ongoing crisis, with some states having reached an initial peak and others still increasing in diagnosed cases and deaths. In addition, there was a regional differentiation within countries in terms of the impact of Covid, with a higher concentration of cases/deaths in the state of New York and California for the U.S. and in the northern regions for Italy.

We strived to ensure comparability between the two country samples. Respondents in Italy (N = 723) were recruited from the same worker pool used in the U.S., thus ensuring roughly comparable socio-economic characteristics for participants from the two countries, and their exposure to identical survey procedures. Monetary incentives were made equivalent in the two countries using the Economist’s Big Mac Index issued in January 2020. We applied quota sampling for geographical residence, gender and age, to ensure equal frequencies in the two countries according to these dimensions. Using the four CDC regions for the U.S. and the two areas of North and South in Italy seemed appropriate to ensure both balanced population sizes and cultural differentiation within each country. Age quotas in Italy were anchored to that obtained in the U.S.. The survey questionnaire was translated from the original English version into Italian by bilingual members of the research team and cross-checked with a third party.

### Demographic characteristics

Basic demographic characteristics of the U.S. and Italian samples are reported in Supplementary Table 1. There were approximately equal numbers of males and females in both samples and a broad range of participants from different demographic groups. The resulting Italian sample had a somewhat lower average age than the U.S. sample, ultimately reflecting lower computer literacy rates of elderly people in Italy compared to the U.S. Respondents in the U.S. sample also had higher education and income levels, reflecting real-life population differences. These demographic variables were controlled for in all analyses.

### Decision task and framing

The full survey is reported in the Supplementary Note SN6 and at https://osf.io/u7zmj/. After participants had responded to a small number of demographic questions (including the respondent’s state or region of residence), the critical decision task was introduced as part of the survey questionnaire. The decision was preceded by a short paragraph reminding participants of the seriousness of the COVID-19 pandemic as a medical and economic crisis. This paragraph provided for the introduction of our framing manipulation. In a Control condition, the content of the paragraph described the consequences of the pandemic in general terms with no mention of any specific geographical region. In the three experimental framing conditions, the content was the same but specific references were inserted to the respondent’s state or region, to the nation (United States or Italy), or to the world, respectively (Full content of the framing paragraph in all four conditions is provided in the Supplementary Note SN6: Section I.2). Participants in the survey were randomly assigned to receive one of the four opening frames.

Participants then received instructions for the critical decision task. After being informed that they would receive a bonus for participating, they were given the opportunity to either keep all the money for themselves or to donate some or all of it to one of three aid organizations providing food, medical and other assistance to those impacted by the pandemic. They were also informed that donations would be doubled by a matching donation from the researchers (For detailed wording, see Supplementary Note SN6: Section I.3).

Respondents were given a comprehension test to be sure they understood the nature of the decision (Supplementary Note SN6: Section I.4). (A participant would be rejected from the study in the event of failure after three test trials, with no further collection of data. The attrition rate due to test failure was 13% in the U.S. and 16% in Italy). They were then asked whether they wanted to make a donation to one of the three listed charities or preferred not to donate. If they chose to make a donation, they specified how much, in any amount up to $5 (€4).

### Predictor variables

In addition to the donation decision variables as our primary dependent measures, we used both exogenous data sources and participants’ responses to items in the survey questionnaire to construct indices for the predictor variables noted in our preregistration.

#### COVID-19 county-level exposure

For our index of environmental exposure to COVID-19, we deviated from the pre-registered analysis plan, which provided for the use of death per 100,000 inhabitants in the participant’s county of residence. Since this measure was not available at the county level in Italy, we opted for using the number of confirmed cases per 100,000 inhabitants instead. (Regressions using the death measure at the state/region level produced qualitatively similar results. See Supplementary Table 3).

We created our environmental exposure measures by utilizing public health data on incidence of COVID-19 cases in each respondent’s district of residence. To create this index, the most immediate geographic unit for which systematic data were available was used. In the U.S. both number of cases and number of deaths were available at the county level. In Italy case data was available at the province (NUTS-3) level, roughly equivalent to county level in the U.S.

In the U.S. we obtained county-level COVID-19 case counts using the data from The New York Times, based on reports from state and local health agencies^59^. This database reports cumulative number of cases and deaths attributed to COVID-19 at different geographical levels daily. This database is updated regularly using reports from state and local health agencies and was made public in late March 2020. We downloaded the cumulative number of cases for each respondent’s county for the day before the survey completion date. We merged to this data set 2019 county population estimates from Census Bureau for a given county (or city) and created a per capita rate of cases per 100,000 inhabitants^60^.

In Italy we obtained province (county)-level COVID-19 case counts from the database maintained by the official site of the national Civil Protection^61^. This database reports cumulative number of cases attributed to COVID-19 at different geographical levels daily. We downloaded cumulative number of cases for each respondent’s county for the day before the survey completion date. We merged to this data set 2019 county population estimates from the Italian National Institute of Statistics (ISTAT) and created a per capita rate of cases per 100,000 inhabitants.

#### Personal exposure to COVID-19

Although our preregistration plan focused on environmental exposure to COVID-19, we also assessed exposure at the individual level. Participants responded to a series of questions asking whether they or others they knew had been diagnosed with COVID-19 and whether they knew anyone who had died as a consequence of contracting the virus. Responses to these questions were used to classify participants as 0 (no contact with diagnosed others) or 1 (self or known others diagnosed or died) as our index of personal exposure to the pandemic (Supplementary Table 1 and Supplementary Note SN6: Section I.6).

#### Region of residence

A dummy variable for region of the country (4 CDC regions in the U.S.; North vs South in Italy) where each participant resided was included in all models to control for main effects of region when looking at effects for county-level exposure data.

#### Political ideology and Conservativism scale

In addition to standard demographic variables (age, sex, 2019 household income, level of education, country of birth), we measured participants’ political orientation on a 5-point scale where 1 means “very Liberal” and 5 means “very Conservative” (see Supplementary Table 1).

#### Social identity scales

We used answers to three questions inquiring about the participant’s attachment, closeness, and perception of being a typical member of the local, national, and international community, to construct a measure of social identity for each level considered in our study^34^ (see Supplementary Note SN6: Section I.8 for item wording). Ratings for each item were made on a 4-point scale and then averaged to create an index of strength of social identity at each level of collectivity.

### Econometric methods

We used a variety of econometric models to analyze the different dependent variables of our study.

#### Hurdle model

The hurdle model^62^ consists of two tiers and enables us to capture the marginal effects for both the decision on whether to donate or not, and how much to donate. The decision of whether to donate or not (extensive margin) was modelled through a Probit model, while the decision on how much to donate (intensive margin) was modeled using a linear specification. While in the pre-analysis plan we specified the use of a double hurdle model, we opted for a single hurdle model because of the paucity of observations at the second hurdle (that is, full donations), and because of (well-known) instability in convergence of double hurdle model. Models were estimated using the *churdle command* in Stata 16.

#### Repeated-observation Tobit model

In addition to hurdle models, we estimated models of donation using a Tobit specification. Unlike hurdle models where two separate indices determined the probability of being a donor and the amount given, in a Tobit setup the parameters of a single latent index of willingness to give were estimated. By introducing controls for charity type we also captured the differential willingness to donate for each type of charity. Binary decision to donate or not were estimated as the probability of this index being untruncated. Conditional on this index being untruncated, amount donated was fitted. Heteroschedasticity-robust standard errors were computed through the bootstrap method with 1,000 repetitions. These models were estimated using xttobit command in Stata 16.

#### Multivariate Tobit and Probit models

We estimated the multivariate model with Correlated Mixed-Process framework using the *cmp* command in Stata16^63^. This framework extends Zellner’s Seemingly Unrelated Regression Equations model^64^ to estimate wide range of flexible systems of equations with correlated errors.

### Effect sizes

Effect sizes have been computed using Cohen’s d. Confidence intervals were obtained from bootstrapped standard errors with 1000 repetitions. Effect sizes for social identities were calculated comparing the group scoring at the highest level (3–4) in the scale with the group scoring at the lowest level (1–2). When Cohen’s d was not applicable, for instance when the independent variable included more than two groups, we reported average marginal effects (Table 1) or regression coefficients (Supplementary Table 8). The *p*-values are derived from econometric analysis and result from two-sided t-tests on the hypothesis that the relevant coefficient is different from 0.

## Supplementary Information

Supplementary Information 1.

## Data Availability

The dataset that supports the findings of this study is available in the project Open Science Foundation at this address: https://osf.io/ns7m5/. Codes used in our statistical analyses are available in the same repository.
