# Supplementary material for: Exposure to COVID-19 is associated with increased altruism, particularly at the local level
Source: Sci Rep. 2021 Sep 23;11:18950. doi: 10.1038/s41598-021-97234-2 (PMC8460816; doi:10.1038/s41598-021-97234-2)
Supplement: Supplementary file 1 — Supplementary Information 1. [file 41598_2021_97234_MOESM1_ESM.pdf]

Exposure to COVID-19 is associated with increased altruism, particularly at the local level.

Supplementary Information

Gianluca Grimalda*
Kiel Institute for the World Economy
Kiellinie 66
24105 Kiel (Germany)
ORCID: 0000-0002-5605-5591

Nancy R. Buchan
Sonoco Department of International Business
University of South Carolina
1014 College Street
Columbia, SC 29205
ORCID: 0000-0002-7611-7227

Orgul D. Ozturk
Department of Economics
University of South Carolina
1014 College Street
Columbia, SC 29205
ORCID: 0000-0001-8446-0271

Adriana C. Pinate
Social Sciences, Gran Sasso Science Institute (GSSI)
67100 L'Aquila, Italy
ORCID: 0000-0002-2262-8371

Giulia Urso
Social Sciences, Gran Sasso Science Institute (GSSI)
67100 L'Aquila, Italy
ORCID: 0000-0003-0696-0363

Marilynn B. Brewer, Professor Emeritus
Department of Psychology
Ohio State University
1835 Neil Avenue
Columbus, OH 43210
ORCID: 0000-0003-3360-003X

Supplementary Material

	United States				Italy				
Measure	Mean / Proportion	Std.Dev.	Min	Max	Mean / Proportion	Std.Dev.	Min	Max	**Question from which variable is based and notes on variable construction**
**Share of Bonus Donated**	0.55	0.31	0.04	1	0.63	0.28	0.03	1	
**Charity Choice**									**Question (Q)16**
Self	36.70%				23.10%				
State/Region	40.99%				32.92%				
National	12.98%				26.56%				
International	9.33%				17.43%				
**Conditional Donation Amount**									**Q17**
State/Region	0.53	0.30	0.04	1	0.56	0.28	0.03	1	
National	0.58	0.32	0.1	1	0.62	0.27	0.03	1	
International	0.62	0.31	0.2	1	0.76	0.28	0.13	1	
**Aggregate Donation**									
State/Region	0.22	0.32	0	1	0.19	0.31	0	1	
National	0.08	0.23	0	1	0.16	0.31	0	1	
International	0.06	0.20	0	1	0.13	0.31	0	1	
**Age**									**Q3**
	36.3	12.85	18	77	32.10	10.48	18	66	Variable expressed in years.
**Female**									**Q2**
	50.2%		0	1	49.7%		0	1	Dummy variable equal to 1 if the respondent is a female and 0 otherwise. Three respondents classified themselves as belonging to the "Other" category, and they have been grouped with males.
**Education Level**									**Q9**
Low	10.1%				4.4%				Categorical variable indicating the level of education of the respondent:<br>Low= High school or less;
Medium	26.2%				70.0%				Medium= Some College or Technical School or Diploma;<br>High= College or more.
High	63.7%				25.6%				We used a five-level scale in econometric analysis.

(Continued)	United States				Italy				
	Mean / Proportion	Std.Dev.	Min	Max	Mean / Proportion	Std.Dev.	Min	Max	
**Place of Residence**									**Q11**
Rural/Village/Town	21.2%				41.9%				Categorical variables indicating the place of residence of the respondent:<br>0= Rural/Village/Town= less than 50,000 inhabitants;<br>1= Small/Medium Metro Area= 50,000 - 1.5 million inhabitants;<br>2= Large Metropolitan Area= larger than 1.5 million inhabitants.
Small/Medium Metro Area	58.3%				41.2%				
Large Metropolitan Area	20.5%				16.9%				
**Rooted**									**Q4 | Q6 | Q7**
	0.74	0.44	0	1	0.89	0.32	0	1	Dummy variable assuming value 1 if the respondent, her mother and her father were all born in the country <U.S. or Italy> and 0 otherwise.
**Geographical Regions**									**Q12**
Northeast	23.6%								Dummy variables indicating the geographical regions where the participant resides.
Midwest	26.6%								
South	24.9%								
West	24.9%								
South Italy					49.9%				
**Conservative scale**									**Q64**
Liberal	51.8%				56.2%				Categorical variable indicating participants' political orientation:<br>In this Table we report the percentage of participants who classified themselves as: "Liberal" or "Very Liberal" (labelled Liberal), "Neither Liberal nor Conservative" (labelled Centrist), or "Conservative" or "Very Conservative" (labelled Conservatives).<br>We used the 5-level scale in the econometric analysis.
Centrist	19.4%				30.7%				
Conservative	28.8%				13.1%				
**Income**									**Q68**
Low	12.0%				42.9%				Categorical variable indicating the 2019 household income bracket levels ranging from (1) $0-9,999/€0 - €5,999 to (10) Over $200,000/Over €100,000. The 10-level scale was used in econometric analysis. In this Table we report the percentage of people falling in:<br>Brackets 1-3 (Labelled Low);<br>Brackets 4-7 (Labelled Middle);<br>Brackets 8-10 (Labelled High).
Middle	62.5%				50.9%				
High	25.5%				6.2%				
**Income Loss**									**Q54**
	38.1%				34.3%				Dummy variable identifying participants who declared to have lost income because of COVID-19.

(Continued)	United States				Italy				
	Mean / Proportion	Std.Dev.	Min	Max	Mean / Proportion	Std.Dev.	Min	Max	
**Priming**									**Q14**
Baseline	24.7%				24.8%				Dummy variable indicating the priming condition randomly assigned to the participant.
State/Region	23.7%				25.2%				
Country	24.7%				24.6%				
World	26.9%				25.5%				
**County-level COVID Exposure**									
Cumulative Number of Cases	683.86	3.11	3.11	3,893.71	373.38	306.05	28.2	1,821.42	The econometric model included the total number of confirmed cases per million of inhabitants in the participant's county of residence the day before participation (expressed in logarithm). In this Table we report the values before log transformation.
**State/Region-level COVID Exposure**									
Cumulative Number of Deaths	37.07	57.82	0	243.47	53.52	55.04	5.04	164.02	The econometric model included the total number of confirmed deaths per million of inhabitants in the participant's state (for U.S.) or region (for Italy) of residence the day before participation (expressed in logarithm). In this Table we report the values before log transformation.
**Personal COVID Exposure**									**Q18a-b | Q19a-b | Q20a-b | Q22**
Diagnosed Self	0.4%				0.6%				The econometric model included a Dummy variable identifying participants who had been infected, or whose family members or acquaintances had been infected, or whose family members or acquaintances had died of COVID-19. This Table reports the percentage of participants who had been infected (labelled "Diagnosed Self"), whose family members or acquaintances had been infected (labelled "Diagnosed Others") or had died (Labelled "Others' Death").
Diagnosed Others:									
Someone you live with	1.7%				2.5%				
Family member or a close friend	16.7%				15.4%				
Acquaintances	26.1%				38.5%				
Others' Death	9.4%				16.7%				
**Social Identity**									**Q37a-b-c | Q38a-b-c | Q39a-b-c**
State/Region	2.39	0.75	1	4	2.64	0.72	1	4	Mean of the participant's score for three items measuring attachment, closeness, and degree to which the participant thinks of herself as a typical member of the state (U.S.)/region (Italy), country or world community. Higher values denote higher social identity.
National	2.54	0.76	1	4	2.93	0.72	1	4	
Global	2.27	0.79	1	4	3.14	0.72	1	4	

(Continued)	United States				Italy				
	Mean / Proportion	Std.Dev.	Min	Max	Mean / Proportion	Std.Dev.	Min	Max	
**Trust in People**									**Q40 | Q41 | Q42 | Q43**
Local Community	3.13	0.90	1	5	2.92	0.89	1	5	Categorical variables indicating the respondent's level of trust in people for each dimension: Local (State/Region), National and International (other countries). The variables entered the econometric model with the same levels used in the corresponding questions:<br>1= I don't trust them at all;<br>5= I completely trust them.
State/Region	3.04	0.86	1	5	2.96	0.89	1	5	
Country	2.72	0.88	1	5	2.65	0.87	1	5	
Other Countries	2.74	0.84	1	5	2.92	0.79	1	5	
**PSYC Vulnerability**									**Q23 | Q24 | Q25**
	0.39	0.38	0	1	0.27	0.33	0	1	Dummy variables were constructed to identify respondents who answered that they worried "always" or "most of the time" (a) that they would get infected, or (b) that their community would get infected from COVID19, or (c) that they agreed "strongly" or "somewhat" to being vulnerable to COVID19. An index of Psychological Vulnerability was then created averaging over these three variables. Higher values denote higher psychological vulnerability.
**Social Relationships Loss**									**Q26b| Q28b | Q32 | Q35b**
	0.53	0.26	0	1	0.42	0.21	0	1	Dummy variables were constructed to identify respondents answering that they had fewer in-person meetings or meetings with organized groups, and performed fewer volunteering activities, after COVID-19, compared to before COVID-19. An index of Social Relationships Loss was created by averaging across these variables. Higher values denote higher social relationship losses.
**Occupation**									**Q67**
Manual workers	29.8%				30.4%				Categorical variable indicating the occupation of the respondent:<br>Manual Workers= Unskilled Labor, Service and Sale Workers, Plant and Machines Operators, and Assemblers, Skilled Agricultural, Forestry and Fishery Workers;<br>Employees/self-employed= Technicians and Associate Professionals; Armed Forces Occupations; Craft and Related Trades Workers;<br>Professionals;<br>Managers.<br>In the econometric analysis Professionals and Managers groups have been merged.
Employees/self-employed	24.1%				39.5%				
Professionals	33.5%				2.0%				
Managers	12.7%				28.1%				

(Continued)	United States				Italy				
	Mean / Proportion	Std.Dev.	Min	Max	Mean / Proportion	Std.Dev.	Min	Max	
**Evaluation of charity characteristics**									**Q68bis2 | Q68bis3 |Q68bis4 | Q68bis5**
**Efficiency**									
No difference					28.6%				Categorical variables identifying participants answering that the most efficient charity in the management of donations operated at state/regional, national, or world level, leaving "No difference" as omitted category.
Regional					45.0%				
National					15.8%				
World					10.6%				
**Goal Effectiveness**									
No difference					15.8%				Ditto with respect to charity effectiveness in achieving goal of relief from COVID-19.
Regional					39.9%				
National					27.2%				
World					17.1%				
**Most Helping Self**									
No difference					10.3%				
Regional					68.5%				Ditto with respect to charity most helping for self in relation to COVID-19.
National					20.0%				
World					1.3%				
**People Most in Need**									
No difference					38.7%				
Regional					3.6%				Ditto with respect to identification of people most in need from COVID-19.
National					10.6%				
World					47.1%				
**Expectation of Others' Contribution**									**Q70bis**
Regional					44.7%				
National					50.3%				Ditto with respect to identification of charity most chosen by other participants.
World					5.0%				
Number of Observations		932				723			
Number of Donors		589				556			

Supplementary Table 1 | Descriptive statistics of samples. The last column indicates the question in the survey from which the variable has been derived, and describes how the variable has been constructed.

	United States				Italy			
	Model 1		Model 2		Model 1		Model 2	
DEP VAR	*P*	*CD*	*P*	*CD*	*P*	*CD*	*P*	*CD*
	(1)	(2)	(3)	(4)	(5)	(6)	(7)	(8)
Age	0.003**	0.003**	0.003**	0.004**	0.002	0.001	0.002	0.001
	[0.001]	[0.001]	[0.001]	[0.001]	[0.001]	[0.002]	[0.001]	[0.002]
Female	0.101***	0.104***	0.097***	0.100**	0.055*	0.049	0.053*	0.046
	[0.023]	[0.031]	[0.023]	[0.031]	[0.026]	[0.031]	[0.026]	[0.031]
Education Level	-0.011	-0.014	-0.012	-0.014	0.024*	0.006	0.023+	0.004
	[0.010]	[0.013]	[0.010]	[0.013]	[0.012]	[0.014]	[0.012]	[0.014]
Small/Medium Metro Area	0.018	0.039	0.02	0.043	-0.008	-0.059+	-0.007	-0.059+
	[0.030]	[0.042]	[0.030]	[0.042]	[0.029]	[0.034]	[0.029]	[0.034]
Large Metropolitan Area	0.021	-0.002	0.016	-0.008	0.016	-0.042	0.017	-0.041
	[0.038]	[0.052]	[0.038]	[0.052]	[0.040]	[0.047]	[0.040]	[0.047]
Rooted	-0.001	-0.039	-0.003	-0.039	0.016	0.007	0.015	0.006
	[0.027]	[0.037]	[0.027]	[0.037]	[0.041]	[0.049]	[0.041]	[0.049]
Midwest	0.007	-0.023	0.011	-0.018				
	[0.033]	[0.044]	[0.033]	[0.044]				
South	-0.01	-0.063	-0.005	-0.057				
	[0.033]	[0.045]	[0.033]	[0.045]				
West	-0.027	-0.043	-0.021	-0.034				
	[0.032]	[0.045]	[0.032]	[0.045]				
South Italy					-0.035	0.017	-0.026	0.028
					[0.029]	[0.034]	[0.029]	[0.034]
Conservative scale	-0.051***	-0.058***	-0.049***	-0.055***	-0.092***	-0.088***	-0.090***	-0.086***
	[0.010]	[0.013]	[0.010]	[0.013]	[0.014]	[0.016]	[0.014]	[0.016]
Income	0.019***	0.015+	0.018**	0.013+	0.006	0.007	0.006	0.007
	[0.006]	[0.008]	[0.006]	[0.008]	[0.007]	[0.008]	[0.007]	[0.008]
Income Lost	-0.026	-0.017	-0.029	-0.021	0.019	0.039	0.019	0.038
	[0.023]	[0.032]	[0.023]	[0.032]	[0.028]	[0.033]	[0.028]	[0.033]
Priming State/Region	-0.057+	-0.012	-0.055+	-0.007	0.03	0.046	0.028	0.042
	[0.033]	[0.044]	[0.033]	[0.044]	[0.036]	[0.043]	[0.036]	[0.044]
Priming Country	-0.059+	-0.069	-0.058+	-0.066	0.034	0.008	0.038	0.012
	[0.033]	[0.044]	[0.033]	[0.044]	[0.038]	[0.045]	[0.038]	[0.045]
Priming World	-0.027	-0.01	-0.024	-0.006	0.027	0.032	0.027	0.032
	[0.033]	[0.043]	[0.033]	[0.043]	[0.036]	[0.044]	[0.036]	[0.044]
County-level COVID Exposure	0.001	0.00	0.001	0.00	0.001	0.003+	0.001	0.003
	[0.002]	[0.003]	[0.002]	[0.003]	[0.002]	[0.002]	[0.002]	[0.002]
Personal COVID Exposure			0.058*	0.077*			0.045+	0.054+
			[0.024]	[0.033]			[0.027]	[0.032]
LR chi2	88.66	48.62	95.04	53.96	83.81	41.23	87.07	44.05
Observations	932	932	932	932	723	723	723	723

Supplementary Table 2a | Econometric analysis of probability of being a donor (*P*) and of conditional donation (*CD*). Estimates of marginal effects from two-part hurdle models are reported. The dependent variable is the share of bonus donated to a charity, without identifying which charity had been chosen. *CD* is the amount donated conditional on being a donor. The first column in each model reports the marginal effects from a Probit model to estimate *P*. The second column in each model reports the marginal effects for *CD*. Variables are defined in Supplementary Table 1. Standard errors are in brackets. *** p<0.001, ** p<0.01, * p<0.05, + p<0.10

	United States		Italy	
	**Model 1**	**Model 2**	**Model 1**	**Model 2**
DEP VAR	*CD*	*CD*	*CD*	*CD*
	(1)	(2)	(3)	(4)
National Charity	0.036+	0.037+	0.044*	0.045*
	[0.019]	[0.019]	[0.020]	[0.020]
International Charity	0.057**	0.057**	0.140***	0.140***
	[0.021]	[0.021]	[0.023]	[0.023]
Age	0.003**	0.003***	0.002+	0.002+
	[0.001]	[0.001]	[0.001]	[0.001]
Female	0.100***	0.096***	0.063*	0.061*
	[0.022]	[0.022]	[0.025]	[0.026]
Education Level	-0.011	-0.011	0.023*	0.022+
	[0.009]	[0.009]	[0.012]	[0.012]
Small/Medium Metro Area	0.017	0.02	-0.005	-0.005
	[0.030]	[0.030]	[0.028]	[0.028]
Large Metropolitan Area	0.02	0.016	0.021	0.021
	[0.038]	[0.037]	[0.039]	[0.039]
Rooted	0.002	0.001	0.028	0.027
	[0.026]	[0.026]	[0.040]	[0.040]
Midwest	0.006	0.009		
	[0.032]	[0.032]		
South	-0.015	-0.01		
	[0.033]	[0.033]		
West	-0.03	-0.024		
	[0.032]	[0.032]		
South Italy			-0.036	-0.028
			[0.028]	[0.028]
Conservative scale	-0.048***	-0.046***	-0.070***	-0.069***
	[0.010]	[0.010]	[0.014]	[0.014]
Income	0.019***	0.018**	0.007	0.007
	[0.006]	[0.006]	[0.007]	[0.007]
Income Lost	-0.028	-0.031	0.021	0.021
	[0.023]	[0.023]	[0.027]	[0.027]
Priming State/Region	-0.055+	-0.053	0.035	0.033
	[0.032]	[0.032]	[0.035]	[0.035]
Priming Country	-0.058+	-0.056+	0.033	0.036
	[0.033]	[0.032]	[0.037]	[0.037]
Priming World	-0.029	-0.026	0.016	0.016
	[0.032]	[0.032]	[0.035]	[0.035]
County-level COVID Exposure	0.001	0.001	0	0
	[0.002]	[0.002]	[0.002]	[0.002]
Personal COVID Exposure		0.057*		0.038
		[0.023]		[0.026]
LR chi2	97.15	103.54	118.36	121.29
Observations	932	932	723	723

Supplementary Table 2b | Econometric analysis of probability of being a donor (*P*) and of conditional donation (*CD*). See Supplementary Table 1 for variable definition and Supplementary Table 2a for model description.

	United States & Italy			
	Model 1		Model 2	
DEP VAR	*P*	*CD*	*P*	*CD*
	(1)	(2)	(3)	(4)
Age	0.003***	0.002*	0.003***	0.002*
	[0.001]	[0.001]	[0.001]	[0.001]
Female	0.081***	0.076***	0.082***	0.076***
	[0.017]	[0.022]	[0.017]	[0.022]
Education Level	0.003	-0.006	0.002	-0.006
	[0.007]	[0.010]	[0.007]	[0.010]
Small/Medium Metro Area	0.007	-0.015	0.006	-0.016
	[0.020]	[0.026]	[0.020]	[0.026]
Large Metropolitan Area	0.005	-0.032	0.003	-0.035
	[0.026]	[0.034]	[0.026]	[0.034]
Rooted	0.005	-0.017	0.003	-0.02
	[0.022]	[0.028]	[0.022]	[0.028]
Conservative scale	-0.063***	-0.065***	-0.050***	-0.051***
	[0.008]	[0.010]	[0.010]	[0.012]
Income	0.013**	0.011*	0.013**	0.011+
	[0.004]	[0.006]	[0.004]	[0.006]
Income Lost	-0.007	0.003	-0.007	0.003
	[0.018]	[0.023]	[0.018]	[0.023]
Priming State/Region	-0.022	0.016	-0.022	0.017
	[0.024]	[0.031]	[0.024]	[0.031]
Priming Country	-0.017	-0.031	-0.016	-0.029
	[0.025]	[0.032]	[0.025]	[0.032]
Priming World	-0.001	0.012	0.000	0.012
	[0.024]	[0.031]	[0.024]	[0.031]
County-level COVID Exposure	0.001	0.002	0.001	0.002
	[0.001]	[0.002]	[0.001]	[0.002]
Personal COVID Exposure	0.053**	0.064**	0.059*	0.068*
	[0.018]	[0.023]	[0.024]	[0.030]
Italy	0.161***	0.149***	0.265***	0.269***
	[0.021]	[0.026]	[0.048]	[0.057]
Conservative X Ita			-0.041*	-0.050*
			[0.017]	[0.023]
Personal Exposure X Ita			-0.012	-0.011
			[0.036]	[0.046]
LR chi2	197.05	116.49	203.22	121.4
Observations	1,655	1,655	1,655	1,655

Supplementary Table 2c | Econometric analysis of probability of being a donor and of conditional donation - Pooled data. The “X” operator indicates an interaction term of the two variables listed. See Supplementary Table 1 for variable definition and Supplementary Table 2a for model description.

	United States		Italy	
	Model 1		Model 1	
DEP VAR	*P*	*CD*	*P*	*CD*
	(1)	(2)	(3)	(4)
Age	0.003**	0.004**	0.002	0.001
	[0.001]	[0.001]	[0.001]	[0.002]
Female	0.097***	0.099**	0.053*	0.046
	[0.023]	[0.031]	[0.026]	[0.031]
Education Level	-0.012	-0.014	0.023+	0.004
	[0.010]	[0.013]	[0.012]	[0.014]
Small/Medium Metro Area	0.023	0.044	-0.004	-0.057+
	[0.030]	[0.041]	[0.029]	[0.034]
Large Metropolitan Area	0.018	-0.007	0.017	-0.034
	[0.038]	[0.052]	[0.039]	[0.046]
Rooted	-0.004	-0.04	0.013	0.002
	[0.027]	[0.037]	[0.041]	[0.049]
Midwest	0.007	-0.021		
	[0.033]	[0.045]		
South	-0.007	-0.058		
	[0.033]	[0.045]		
West	-0.023	-0.036		
	[0.033]	[0.045]		
South Italy			0.001	0.042
			[0.038]	[0.044]
Conservative scale	-0.049***	-0.055***	-0.090***	-0.087***
	[0.010]	[0.013]	[0.014]	[0.016]
Income	0.018**	0.013+	0.006	0.008
	[0.006]	[0.008]	[0.007]	[0.008]
Income Lost	-0.029	-0.021	0.016	0.037
	[0.023]	[0.032]	[0.028]	[0.033]
Priming State/Region	-0.055+	-0.006	0.028	0.044
	[0.033]	[0.044]	[0.036]	[0.044]
Priming Country	-0.056+	-0.065	0.042	0.02
	[0.033]	[0.044]	[0.038]	[0.045]
Priming World	-0.024	-0.006	0.031	0.037
	[0.033]	[0.043]	[0.036]	[0.044]
State/Region-level COVID Exposure	-0.001	-0.001	0.019	0.018
	[0.002]	[0.002]	[0.016]	[0.019]
Personal COVID Exposure	0.058*	0.077*	0.042	0.055+
	[0.024]	[0.033]	[0.027]	[0.032]
LR chi2	94.98	54.06	85.62	42.84
Observations	932	932	723	723

Supplementary Table 3 | Econometric analysis of probability of being a donor and of conditional donation - U.S. and Italy. Death count. See notes to Supplementary Table 2a. Environmental COVID-19 exposure is measured by the number of deaths in the state (for U.S.) or region (for Italy), where the participant resides. See Supplementary Table 1 for variable definition and Supplementary Table 2a for model description.

	United States	Italy	United States & Italy	
	Model 1	Model 1	Model 1	Model 2
DEP VAR	*AD*	*AD*	*AD*	*AD*
	(1)	(2)	(3)	(4)
National Charity	-0.857***	-0.168+	-0.566***	-1.677***
	[0.078]	[0.096]	[0.060]	[0.186]
International Charity	-1.059***	-0.460***	-0.813***	-1.838***
	[0.083]	[0.095]	[0.063]	[0.210]
Age	0.004*	0.002	0.004*	0.004**
	[0.002]	[0.002]	[0.001]	[0.001]
Female	0.165***	0.077+	0.128***	0.129***
	[0.045]	[0.041]	[0.031]	[0.031]
Education Level	-0.026	0.025	-0.002	-0.003
	[0.018]	[0.021]	[0.014]	[0.013]
Small/Medium Metro Area	0.051	-0.044	-0.001	-0.001
	[0.060]	[0.046]	[0.035]	[0.038]
Large Metropolitan Area	0.018	-0.016	-0.01	-0.012
	[0.072]	[0.068]	[0.044]	[0.046]
Rooted	-0.042	0.009	-0.012	-0.018
	[0.052]	[0.068]	[0.041]	[0.041]
Midwest	0.003			
	[0.060]			
South	-0.017			
	[0.065]			
West	-0.037			
	[0.063]			
South Italy		0.001		
		[0.045]		
Conservative scale	-0.098***	-0.159***	-0.123***	-0.122***
	[0.019]	[0.025]	[0.015]	[0.016]
Income	0.024*	0.008	0.019*	0.018*
	[0.011]	[0.012]	[0.008]	[0.008]
Income Lost	-0.039	0.038	-0.005	-0.003
	[0.045]	[0.045]	[0.034]	[0.032]
Priming State/Region	-0.065	0.048	-0.018	-0.017
	[0.060]	[0.060]	[0.042]	[0.041]
Priming Country	-0.095	0.046	-0.033	-0.032
	[0.064]	[0.062]	[0.043]	[0.045]
Priming World	-0.025	0.056	0.008	0.011
	[0.060]	[0.062]	[0.041]	[0.043]
County Level COVID Exposure	0.000	0.003	0.003	0.002
	[0.004]	[0.003]	[0.002]	[0.002]
Personal COVID Exposure	0.104*	0.078+	0.090**	0.091**
	[0.047]	[0.045]	[0.032]	[0.031]
Italy			0.264***	
			[0.040]	
Local level X Ita				-0.163*
				[0.069]
Country level X Ita				0.598***
				[0.087]
World level X Ita				0.544***
				[0.103]
Constant	-0.335*	-0.519**	-0.554***	-0.194
	[0.150]	[0.160]	[0.107]	[0.150]
Observations	2,796	2,169	4,965	4,965
Number of ID	932	723	1655	1655

Supplementary Table 4 | Econometric analysis of Aggregate Donations with panel model - US, Italy and pooled country data. Coefficient estimates from a Tobit model of donation using charity choice as the panel dimension. Heteroskedasticity-robust standard errors were computed through the bootstrap method with 1,000 repetitions. The tests on the null hypotheses that charity choice dummy coefficients were equal to each other were either taken from the Table (for pairwise comparisons including the local charity) or from a post-estimation chi2 test (not reported in the Table). The “X” operator indicates an interaction term of the two variables listed. See Supplementary Table 1 for variables’ definition.

	United States									Italy								
	Model 1			Model 2			Model 3			Model 1			Model 2			Model 3		
DEP VAR: AD	State	Country	World	State	Country	World	State	Country	World	Region	Country	World	Region	Country	World	Region	Country	World
	(1)	(2)	(3)	(4)	(5)	(6)	(7)	(8)	(9)	(10)	(11)	(12)	(13)	(14)	(15)	(16)	(17)	(18)
Age	0.009***	-0.011*	0.00	0.008***	-0.011*	0.002	0.009***	-0.011*	0.00	0.009**	-0.011**	0.002	0.007*	-0.010*	0.005	0.007*	-0.011*	0.005
	[0.002]	[0.005]	[0.006]	[0.002]	[0.005]	[0.006]	[0.002]	[0.005]	[0.006]	[0.003]	[0.004]	[0.006]	[0.003]	[0.004]	[0.006]	[0.003]	[0.004]	[0.006]
Female	0.131**	0.124	0.086	0.138**	0.117	0.07	0.117*	0.163	0.13	0.144*	0.06	-0.127	0.165*	0.043	-0.142	0.173*	0.046	-0.119
	[0.049]	[0.108]	[0.135]	[0.048]	[0.107]	[0.133]	[0.048]	[0.108]	[0.135]	[0.067]	[0.081]	[0.123]	[0.067]	[0.081]	[0.121]	[0.069]	[0.084]	[0.124]
Education Level	-0.004	-0.078+	0.017	-0.006	-0.075+	0.019	-0.013	-0.056	0.015	0.005	0.019	0.027	0.024	-0.001	0.028	0.017	-0.002	0.009
	[0.020]	[0.044]	[0.056]	[0.020]	[0.044]	[0.055]	[0.022]	[0.049]	[0.063]	[0.030]	[0.038]	[0.054]	[0.030]	[0.038]	[0.053]	[0.032]	[0.040]	[0.056]
Small/Medium Metro Area	0.065	-0.056	0.169	0.052	-0.067	0.205	0.063	-0.014	0.212	0.019	-0.115	0.007	0.022	-0.139	0.038	0.02	-0.151	0.059
	[0.065]	[0.142]	[0.187]	[0.064]	[0.142]	[0.187]	[0.064]	[0.143]	[0.188]	[0.073]	[0.091]	[0.134]	[0.071]	[0.090]	[0.131]	[0.073]	[0.093]	[0.133]
Large Metropolitan Area	0.026	0.00	0.00	-0.001	-0.014	0.065	0.025	0.04	0.05	-0.1	0.185	-0.202	-0.073	0.124	-0.176	-0.064	0.089	-0.063
	[0.080]	[0.174]	[0.229]	[0.079]	[0.174]	[0.228]	[0.079]	[0.177]	[0.230]	[0.103]	[0.116]	[0.189]	[0.101]	[0.115]	[0.185]	[0.105]	[0.121]	[0.191]
Rooted	0.013	0.047	-0.305*	0.003	0.025	-0.305*	0.002	-0.047	-0.350*	-0.067	0.487**	-0.373*	-0.068	0.457**	-0.339*	-0.030	0.448**	-0.372*
	[0.058]	[0.124]	[0.148]	[0.057]	[0.123]	[0.145]	[0.057]	[0.123]	[0.148]	[0.103]	[0.153]	[0.174]	[0.101]	[0.151]	[0.168]	[0.103]	[0.153]	[0.170]
Midwest	-0.009	-0.044	0.109	0.033	-0.07	0.019	0.027	-0.097	-0.031									
	[0.068]	[0.159]	[0.192]	[0.067]	[0.158]	[0.191]	[0.067]	[0.157]	[0.193]									
South	-0.117+	0.212	0.069	-0.097	0.166	-0.014	-0.095	0.119	-0.019									
	[0.071]	[0.154]	[0.196]	[0.069]	[0.152]	[0.194]	[0.070]	[0.152]	[0.195]									
West	-0.094	0.093	0.091	-0.098	0.112	0.098	-0.118+	0.09	0.094									
	[0.070]	[0.156]	[0.195]	[0.069]	[0.155]	[0.192]	[0.069]	[0.155]	[0.195]									
South Italy										-0.049	0.057	-0.015	-0.042	0.051	-0.012	-0.047	0.095	-0.035
										[0.074]	[0.090]	[0.133]	[0.072]	[0.089]	[0.129]	[0.076]	[0.094]	[0.135]
Conservative scale	-0.032	-0.095*	-0.183**	-0.045+	-0.121*	-0.126+	-0.029	-0.102+	-0.111	0.084*	-0.051	-0.627***	0.044	-0.062	-0.511***	0.034	-0.046	-0.516***
	[0.021]	[0.047]	[0.063]	[0.023]	[0.054]	[0.069]	[0.024]	[0.056]	[0.071]	[0.036]	[0.046]	[0.088]	[0.037]	[0.048]	[0.084]	[0.038]	[0.049]	[0.086]
Income	0.027*	0.012	0.00	0.021+	0.015	0.013	0.016	0.018	0.01	0.004	0.044*	-0.042	-0.007	0.044*	-0.02	-0.011	0.044*	-0.014
	[0.012]	[0.026]	[0.033]	[0.012]	[0.026]	[0.032]	[0.013]	[0.027]	[0.034]	[0.018]	[0.021]	[0.032]	[0.018]	[0.021]	[0.032]	[0.018]	[0.022]	[0.032]
Income Lost	-0.082	0.058	0.057	-0.085+	0.035	0.058	-0.085+	0.027	0.07	0.014	0.129	-0.063	0.021	0.111	-0.073	0.016	0.134	-0.053
	[0.050]	[0.108]	[0.135]	[0.050]	[0.108]	[0.134]	[0.050]	[0.109]	[0.136]	[0.072]	[0.087]	[0.132]	[0.071]	[0.086]	[0.128]	[0.072]	[0.089]	[0.131]
Priming State/Region	-0.022	-0.071	-0.134	-0.033	-0.078	-0.113	-0.009	-0.094	-0.132	0.169+	-0.051	-0.069	0.198*	-0.098	-0.099	0.221*	-0.095	-0.131
	[0.068]	[0.151]	[0.196]	[0.067]	[0.150]	[0.195]	[0.068]	[0.151]	[0.198]	[0.094]	[0.110]	[0.179]	[0.092]	[0.109]	[0.174]	[0.095]	[0.112]	[0.180]
Priming Country	-0.091	-0.048	-0.083	-0.09	-0.064	-0.045	-0.07	-0.06	-0.039	0.118	-0.087	0.084	0.128	-0.096	0.039	0.122	-0.122	0.049
	[0.069]	[0.149]	[0.194]	[0.067]	[0.148]	[0.192]	[0.068]	[0.148]	[0.193]	[0.096]	[0.113]	[0.176]	[0.094]	[0.111]	[0.171]	[0.098]	[0.116]	[0.177]
Priming World	-0.06	-0.121	0.228	-0.067	-0.12	0.256	-0.039	-0.119	0.266	0.125	-0.268*	0.372*	0.133	-0.287*	0.331*	0.155+	-0.297*	0.342*
	[0.067]	[0.147]	[0.180]	[0.066]	[0.146]	[0.179]	[0.066]	[0.145]	[0.181]	[0.094]	[0.117]	[0.168]	[0.092]	[0.115]	[0.163]	[0.094]	[0.119]	[0.167]

(Continued)					United States									Italy				
	(1)	(2)	(3)	(4)	(5)	(6)	(7)	(8)	(9)	(10)	(11)	(12)	(13)	(14)	(15)	(16)	(17)	(18)
County-level COVID Exposure	0.001	-0.008	0.006	0.001	-0.006	0.007	0.00	-0.009	0.006	-0.003	0.006	0.013	-0.002	0.003	0.014	-0.003	0.003	0.014
	[0.004]	[0.009]	[0.013]	[0.004]	[0.009]	[0.013]	[0.004]	[0.009]	[0.013]	[0.004]	[0.005]	[0.009]	[0.004]	[0.005]	[0.009]	[0.004]	[0.005]	[0.009]
Personal COVID Exposure	0.087+	0.076	0.085	0.065	0.069	0.051	0.07	0.023	0.04	0.069	-0.089	0.227+	0.056	-0.059	0.186	0.06	-0.059	0.169
	[0.051]	[0.111]	[0.139]	[0.050]	[0.112]	[0.139]	[0.051]	[0.113]	[0.142]	[0.068]	[0.083]	[0.127]	[0.067]	[0.083]	[0.125]	[0.070]	[0.086]	[0.128]
Local Social Identity				0.214***	-0.208**	-0.270**	0.157***	-0.203*	-0.310**				0.298***	-0.271***	-0.132	0.308***	-0.296***	-0.178
				[0.035]	[0.080]	[0.102]	[0.039]	[0.089]	[0.113]				[0.055]	[0.069]	[0.100]	[0.063]	[0.080]	[0.115]
National Social Identity				-0.021	0.202*	-0.037	-0.012	0.273**	-0.086				-0.119*	0.315***	-0.218*	-0.145*	0.349***	-0.252*
				[0.038]	[0.088]	[0.108]	[0.042]	[0.096]	[0.120]				[0.055]	[0.071]	[0.101]	[0.063]	[0.082]	[0.115]
Global Social Identity				-0.053	0.093	0.292**	-0.071*	0.065	0.270**				-0.095*	-0.049	0.489***	-0.09	-0.039	0.461***
				[0.033]	[0.069]	[0.089]	[0.036]	[0.074]	[0.094]				[0.048]	[0.060]	[0.102]	[0.055]	[0.068]	[0.113]
Trust People from State/Region							0.064	-0.135	0.126							-0.042	0.001	0.159
							[0.045]	[0.098]	[0.127]							[0.058]	[0.071]	[0.108]
Trust People from Country							-0.075+	-0.13	0.014							0.011	-0.021	0.105
							[0.040]	[0.089]	[0.114]							[0.057]	[0.071]	[0.105]
Trust People from Other Country							0.034	0.219**	0.11							-0.04	0.061	-0.006
							[0.037]	[0.084]	[0.106]							[0.053]	[0.066]	[0.103]
Trust Local Community							0.079*	0.142	-0.011							0.033	0.065	-0.168
							[0.040]	[0.089]	[0.113]							[0.056]	[0.071]	[0.104]
PSYC Vulnerability							0.081	-0.094	-0.092							0.018	-0.106	-0.219
							[0.068]	[0.154]	[0.188]							[0.104]	[0.133]	[0.198]
Social Relationships Loss							0.036	0.045	-0.011							0.252	-0.352+	-0.006
							[0.097]	[0.209]	[0.266]							[0.155]	[0.197]	[0.285]
Employees/self-employed							0.014	-0.163	-0.078							0.117	-0.003	0.082
							[0.068]	[0.151]	[0.193]							[0.086]	[0.106]	[0.157]
Professionals/Managers							0.013	-0.152	0.028							0.083	0.023	0.013
							[0.067]	[0.141]	[0.190]							[0.091]	[0.109]	[0.160]
Constant	-0.530**	-0.406	-1.218**	-0.754***	-0.54	-1.427**	-1.012***	-0.926*	-1.724**	-0.896***	-0.679*	0.698	-0.924**	-0.621+	-0.295	-0.931**	-0.916*	-0.125
	[0.163]	[0.339]	[0.457]	[0.185]	[0.392]	[0.525]	[0.206]	[0.442]	[0.592]	[0.243]	[0.309]	[0.432]	[0.294]	[0.377]	[0.546]	[0.314]	[0.412]	[0.592]
LR chi2		100.34			174.58			211.93			178.66			270.91			282.5	
Observations		932			932			912			723			723			686	

Supplementary Table 5a | Econometric analysis of Aggregate Donations (*AD*). We fit multivariate Tobit models to estimate *AD* for each of the three charities. *AD* is the overall amount of donation to each charity, combining both the extensive margin (which charity is chosen) and the intensive margin (Conditional Donations to each charity). Variables are defined in Supplementary Table 1. Standard errors are in brackets. *** p<0.001, ** p<0.01, * p<0.05, + p<0.10.

United States & Italy												
	**Model 1**			**Model 2**			**Model 3**			**Model 4**		
DEP VAR: *AD*	State/Region	Country	World	State/Region	Country	World	State/Region	Country	World	State/Region	Country	World
	(1)	(2)	(3)	(4)	(5)	(6)	(7)	(8)	(9)	(10)	(11)	(12)
Age	0.009***	-0.011***	0.00	0.008***	-0.011***	0.003	0.008***	-0.011***	0.003	0.008***	-0.011***	0.003
	[0.002]	[0.003]	[0.004]	[0.002]	[0.003]	[0.004]	[0.002]	[0.003]	[0.004]	[0.002]	[0.003]	[0.004]
Female	0.138***	0.088	-0.039	0.150***	0.072	-0.062	0.153***	0.072	-0.049	0.136***	0.088	-0.031
	[0.040]	[0.065]	[0.091]	[0.039]	[0.065]	[0.090]	[0.039]	[0.064]	[0.088]	[0.040]	[0.066]	[0.091]
Education Level	0.003	-0.033	0.032	0.007	-0.039	0.024	0.009	-0.04	0.025	-0.003	-0.031	0.021
	[0.017]	[0.029]	[0.039]	[0.017]	[0.028]	[0.038]	[0.017]	[0.028]	[0.038]	[0.018]	[0.031]	[0.041]
Small/Medium Metro Area	0.028	-0.073	0.07	0.026	-0.089	0.097	0.029	-0.093	0.084	0.033	-0.089	0.113
	[0.047]	[0.077]	[0.107]	[0.046]	[0.076]	[0.106]	[0.046]	[0.076]	[0.104]	[0.047]	[0.077]	[0.107]
Large Metropolitan Area	-0.047	0.14	-0.164	-0.054	0.103	-0.12	-0.049	0.094	-0.106	-0.033	0.096	-0.073
	[0.061]	[0.094]	[0.141]	[0.060]	[0.094]	[0.138]	[0.060]	[0.094]	[0.136]	[0.061]	[0.097]	[0.141]
Rooted	-0.018	0.211*	-0.332**	-0.015	0.179*	-0.334**	-0.013	0.178*	-0.326**	0.001	0.128	-0.349**
	[0.051]	[0.089]	[0.111]	[0.050]	[0.088]	[0.108]	[0.050]	[0.088]	[0.106]	[0.051]	[0.089]	[0.109]
Conservative scale	0.001	-0.079*	-0.363***	-0.01	-0.106**	-0.282***	-0.043+	-0.107*	-0.131*	-0.009	-0.089*	-0.284***
	[0.019]	[0.032]	[0.052]	[0.020]	[0.035]	[0.052]	[0.025]	[0.047]	[0.064]	[0.020]	[0.036]	[0.053]
Income	0.020+	0.028+	-0.021	0.013	0.031+	-0.007	0.012	0.029+	-0.002	0.007	0.031+	-0.005
	[0.010]	[0.016]	[0.023]	[0.010]	[0.016]	[0.022]	[0.010]	[0.016]	[0.022]	[0.010]	[0.017]	[0.023]
Income Lost	-0.044	0.101	-0.007	-0.039	0.077	-0.023	-0.041	0.078	-0.008	-0.044	0.085	-0.018
	[0.041]	[0.067]	[0.094]	[0.041]	[0.067]	[0.093]	[0.041]	[0.067]	[0.091]	[0.041]	[0.068]	[0.094]
Priming State/Region	0.051	-0.042	-0.114	0.056	-0.069	-0.117	0.055	-0.072	-0.111	0.083	-0.073	-0.137
	[0.055]	[0.089]	[0.133]	[0.055]	[0.089]	[0.131]	[0.055]	[0.089]	[0.129]	[0.055]	[0.090]	[0.133]
Priming Country	-0.006	-0.059	-0.01	-0.002	-0.074	-0.002	-0.006	-0.074	-0.014	0.013	-0.092	0.007
	[0.056]	[0.090]	[0.131]	[0.055]	[0.089]	[0.128]	[0.055]	[0.089]	[0.126]	[0.056]	[0.092]	[0.130]
Priming World	0.007	-0.179*	0.287*	0.008	-0.194*	0.295*	0.007	-0.195*	0.288*	0.037	-0.203*	0.309*
	[0.055]	[0.091]	[0.123]	[0.054]	[0.090]	[0.121]	[0.054]	[0.090]	[0.119]	[0.054]	[0.092]	[0.122]
County-level COVID Exposure	-0.001	0.001	0.011	-0.001	0.001	0.012	0.000	0.001	0.012+	-0.002	0.001	0.012+
	[0.003]	[0.005]	[0.007]	[0.003]	[0.005]	[0.007]	[0.003]	[0.005]	[0.007]	[0.003]	[0.005]	[0.007]
Personal COVID Exposure	0.091*	-0.042	0.175+	0.072+	-0.022	0.139	0.082	0.026	0.066	0.070+	-0.041	0.133
	[0.041]	[0.066]	[0.092]	[0.040]	[0.066]	[0.092]	[0.053]	[0.097]	[0.130]	[0.041]	[0.068]	[0.093]
Italy	-0.039	0.392***	0.487***	-0.031	0.316***	0.312*	-0.016	0.453	0.727	0.002	0.275**	0.315*
	[0.049]	[0.079]	[0.114]	[0.054]	[0.086]	[0.123]	[0.236]	[0.375]	[0.531]	[0.058]	[0.094]	[0.134]
Local Social Identity				0.245***	-0.239***	-0.225**	0.222***	-0.200**	-0.244**	0.197***	-0.233***	-0.260***
				[0.029]	[0.051]	[0.070]	[0.037]	[0.069]	[0.093]	[0.033]	[0.058]	[0.079]
National Social Identity				-0.075*	0.253***	-0.084	-0.026	0.174*	-0.034	-0.069*	0.303***	-0.118
				[0.031]	[0.054]	[0.073]	[0.041]	[0.076]	[0.101]	[0.035]	[0.061]	[0.082]

(Continued)	**United States & Italy**											
	(1)	(2)	(3)	(4)	(5)	(6)	(7)	(8)	(9)	(10)	(11)	(12)
Global Social Identity				-0.061*	0.025	0.374***	-0.051	0.092	0.282***	-0.073*	0.009	0.346***
				[0.028]	[0.045]	[0.066]	[0.035]	[0.061]	[0.082]	[0.030]	[0.049]	[0.071]
Trust People from State/Region										0.029	-0.05	0.157+
										[0.035]	[0.058]	[0.083]
Trust People from Country										-0.046	-0.064	0.059
										[0.033]	[0.054]	[0.076]
Trust People from Other Country										0.007	0.126*	0.057
										[0.031]	[0.051]	[0.073]
Trust Local Community										0.070*	0.068	-0.115
										[0.033]	[0.055]	[0.076]
PSYC Vulnerability										0.064	-0.033	-0.2
										[0.057]	[0.098]	[0.134]
Social Relationships Loss										0.104	-0.151	0.011
										[0.083]	[0.140]	[0.194]
Employees/self-employed										0.064	-0.046	-0.012
										[0.053]	[0.087]	[0.121]
Professionals/Managers										0.06	-0.057	-0.003
										[0.053]	[0.085]	[0.121]
Conservative X Ita							0.084*	0.027	-0.386***			
							[0.042]	[0.069]	[0.104]			
Personal Exposure X Ita							-0.025	-0.094	0.123			
							[0.080]	[0.131]	[0.180]			
Local Social Identity X Ita							0.049	-0.103	0.107			
							[0.061]	[0.101]	[0.137]			
National Social Identity X Ita							[0.061]	-0.092	0.185+	-0.203		
							[0.064]	[0.107]	[0.144]			
Global Social Identity X Ita							[0.064]	-0.032	-0.154+	0.222+		
							[0.056]	[0.089]	[0.130]			
Constant	-0.691***	-0.595**	-0.538+	-0.870***	-0.594*	-0.991**	-0.877***	-0.641*	-1.149**	-1.029***	-0.817**	-1.113**
	[0.132]	[0.211]	[0.295]	[0.151]	[0.248]	[0.347]	[0.168]	[0.287]	[0.395]	[0.168]	[0.279]	[0.391]
LR chi2		288			447.01			488.17			469.34	
Observations		1,655			1,655			1,655			1,598	

Supplementary Table 5b | Econometric analysis of Aggregate Donations (*AD*) - Pooled observations. We apply the same multivariate Tobit specification used in Supplementary Table 5a to the pooled dataset of U.S. and Italy observations. The "X" operator indicates an interaction term of the two variables listed. See notes to Supplementary Table 5a and Supplementary Table 1 for variables' definition. *** p<0.001, ** p<0.01, * p<0.05, + p<0.10

	United States									Italy								
	Model 1			Model 2			Model 3			Model 1			Model 2			Model 3		
DEP VAR: *P*	State	Country	World	State	Country	World	State	Country	World	Region	Country	World	Region	Country	World	Region	Country	World
	(1)	(2)	(3)	(4)	(5)	(6)	(7)	(8)	(9)	(10)	(11)	(12)	(13)	(14)	(15)	(16)	(17)	(18)
Age	0.020***	-0.004	0.01	0.019***	-0.005	0.008	0.020***	-0.004	0.01	0.013+	-0.013	0.002	0.011	-0.012	0.003	0.012	-0.011	0.005
	[0.005]	[0.007]	[0.007]	[0.005]	[0.007]	[0.007]	[0.006]	[0.007]	[0.008]	[0.008]	[0.008]	[0.009]	[0.008]	[0.008]	[0.010]	[0.008]	[0.009]	[0.010]
Female	0.390**	0.347*	0.287+	0.407**	0.344*	0.276	0.394**	0.412**	0.355*	0.280+	0.231	-0.013	0.294+	0.184	-0.062	0.347*	0.226	-0.015
	[0.126]	[0.153]	[0.166]	[0.128]	[0.154]	[0.169]	[0.132]	[0.160]	[0.174]	[0.158]	[0.164]	[0.184]	[0.161]	[0.167]	[0.189]	[0.170]	[0.177]	[0.200]
Education Level	-0.028	-0.118+	-0.008	-0.034	-0.120+	-0.01	-0.041	-0.102	-0.011	0.006	0.039	0.026	0.039	0.014	0.031	0.007	-0.011	-0.013
	[0.053]	[0.063]	[0.069]	[0.054]	[0.064]	[0.070]	[0.061]	[0.072]	[0.081]	[0.072]	[0.075]	[0.082]	[0.074]	[0.077]	[0.085]	[0.079]	[0.082]	[0.091]
Small/Medium Metro Area	0.187	0.029	0.244	0.171	0.01	0.277	0.204	0.083	0.302	-0.19	-0.362*	-0.209	-0.185	-0.404*	-0.17	-0.199	-0.436*	-0.157
	[0.166]	[0.202]	[0.227]	[0.169]	[0.204]	[0.233]	[0.172]	[0.211]	[0.238]	[0.171]	[0.180]	[0.199]	[0.174]	[0.184]	[0.206]	[0.182]	[0.192]	[0.215]
Large Metropolitan Area	0.013	-0.04	-0.027	-0.057	-0.089	-0.001	-0.03	-0.04	-0.025	-0.28	-0.006	-0.352	-0.249	-0.096	-0.343	-0.164	-0.037	-0.129
	[0.204]	[0.248]	[0.278]	[0.207]	[0.252]	[0.285]	[0.212]	[0.261]	[0.291]	[0.243]	[0.241]	[0.281]	[0.247]	[0.246]	[0.290]	[0.264]	[0.263]	[0.309]
Rooted	-0.076	-0.05	-0.383*	-0.11	-0.091	-0.415*	-0.181	-0.244	-0.531**	-0.093	0.582*	-0.406	-0.088	0.580*	-0.392	-0.024	0.597*	-0.417
	[0.148]	[0.177]	[0.182]	[0.150]	[0.179]	[0.185]	[0.157]	[0.185]	[0.192]	[0.246]	[0.284]	[0.270]	[0.249]	[0.287]	[0.275]	[0.257]	[0.295]	[0.283]
Midwest	-0.095	-0.093	0.09	0.009	-0.082	0.048	-0.041	-0.167	-0.053									
	[0.179]	[0.225]	[0.239]	[0.182]	[0.228]	[0.245]	[0.186]	[0.234]	[0.251]									
South	-0.350+	0.111	-0.034	-0.325+	0.08	-0.101	-0.344+	0.013	-0.12									
	[0.182]	[0.218]	[0.241]	[0.185]	[0.220]	[0.246]	[0.189]	[0.226]	[0.252]									
West	-0.206	0.003	0.05	-0.218	0.033	0.067	-0.273	-0.018	0.039									
	[0.181]	[0.223]	[0.241]	[0.184]	[0.225]	[0.245]	[0.190]	[0.232]	[0.253]									
South Italy										0.04	0.22	0.068	0.021	0.205	0.047	0.047	0.276	0.048
										[0.173]	[0.179]	[0.198]	[0.176]	[0.182]	[0.203]	[0.189]	[0.196]	[0.218]
Conservative scale	-0.154**	-0.22**	-0.30***	-0.173**	-0.25**	-0.26**	-0.125+	-0.199*	-0.213*	-0.17+	-0.33***	-0.97***	-0.209*	-0.34***	-0.89***	-0.235*	-0.33**	-0.92***
	[0.054]	[0.067]	[0.075]	[0.062]	[0.077]	[0.086]	[0.067]	[0.082]	[0.091]	[0.087]	[0.093]	[0.117]	[0.092]	[0.098]	[0.122]	[0.096]	[0.103]	[0.127]
Income	0.055+	0.038	0.03	0.039	0.035	0.036	0.031	0.035	0.02	0.026	0.07	-0.019	0.007	0.073+	0.002	-0.007	0.063	-0.001
	[0.032]	[0.037]	[0.040]	[0.032]	[0.038]	[0.041]	[0.034]	[0.040]	[0.044]	[0.042]	[0.043]	[0.048]	[0.043]	[0.044]	[0.050]	[0.045]	[0.046]	[0.052]
Income Lost	-0.134	0.032	0.026	-0.15	0.008	0.029	-0.189	-0.018	0.014	0.147	0.28	0.084	0.147	0.255	0.063	0.132	0.27	0.078
	[0.129]	[0.155]	[0.167]	[0.132]	[0.157]	[0.171]	[0.137]	[0.163]	[0.177]	[0.171]	[0.178]	[0.199]	[0.175]	[0.181]	[0.203]	[0.182]	[0.189]	[0.212]
Priming State/Region	-0.010	-0.104	-0.088	-0.01	-0.1	-0.051	0.065	-0.09	-0.042	0.343	0.069	0.058	0.424+	0.024	0.048	0.485*	0.052	0.032
	[0.179]	[0.217]	[0.241]	[0.182]	[0.220]	[0.247]	[0.187]	[0.228]	[0.255]	[0.221]	[0.225]	[0.263]	[0.225]	[0.229]	[0.270]	[0.234]	[0.239]	[0.285]
Priming Country	-0.261	-0.206	-0.241	-0.252	-0.212	-0.193	-0.206	-0.185	-0.161	0.124	-0.058	0.118	0.162	-0.06	0.09	0.126	-0.122	0.083
	[0.177]	[0.212]	[0.238]	[0.179]	[0.215]	[0.243]	[0.184]	[0.222]	[0.249]	[0.222]	[0.226]	[0.260]	[0.225]	[0.229]	[0.266]	[0.236]	[0.241]	[0.280]
Priming World	-0.085	-0.134	0.211	-0.081	-0.114	0.273	-0.011	-0.085	0.32	0.252	-0.236	0.490*	0.279	-0.272	0.493+	0.364	-0.229	0.583*
	[0.174]	[0.211]	[0.224]	[0.177]	[0.213]	[0.229]	[0.182]	[0.218]	[0.235]	[0.219]	[0.229]	[0.249]	[0.221]	[0.233]	[0.255]	[0.231]	[0.244]	[0.268]

(Continued)	United States									Italy								
	(1)	(2)	(3)	(4)	(5)	(6)	(7)	(8)	(9)	(10)	(11)	(12)	(13)	(14)	(15)	(16)	(17)	(18)
County-level COVID Exposure	0.002	-0.009	0.003	0.002	-0.007	0.004	0	-0.012	0	0.006	0.018+	0.02	0.008	0.016	0.022+	0.006	0.015	0.023+
	[0.011]	[0.013]	[0.016]	[0.012]	[0.013]	[0.016]	[0.012]	[0.013]	[0.016]	[0.009]	[0.010]	[0.012]	[0.010]	[0.010]	[0.013]	[0.010]	[0.011]	[0.013]
Personal COVID Exposure	0.273*	0.23	0.271	0.192	0.17	0.179	0.188	0.101	0.156	0.246	0.067	0.422*	0.203	0.076	0.362+	0.226	0.095	0.357+
	[0.133]	[0.160]	[0.172]	[0.137]	[0.164]	[0.178]	[0.142]	[0.171]	[0.185]	[0.161]	[0.167]	[0.188]	[0.165]	[0.171]	[0.195]	[0.173]	[0.180]	[0.204]
Local Social Identity				0.512***	-0.068	-0.09	0.349**	-0.15	-0.215				0.486***	-0.324*	-0.108	0.488**	-0.388*	-0.206
				[0.094]	[0.115]	[0.128]	[0.107]	[0.132]	[0.144]				[0.130]	[0.138]	[0.157]	[0.154]	[0.163]	[0.185]
National Social Identity				-0.038	0.232+	-0.011	-0.008	0.336*	-0.035				-0.15	0.444**	-0.225	-0.17	0.514**	-0.251
				[0.101]	[0.124]	[0.136]	[0.112]	[0.139]	[0.153]				[0.132]	[0.141]	[0.157]	[0.156]	[0.166]	[0.184]
Global Social Identity				0.028	0.206*	0.405***	-0.049	0.118	0.334**				-0.028	0.064	0.672***	-0.081	0.029	0.611***
				[0.087]	[0.101]	[0.110]	[0.096]	[0.110]	[0.119]				[0.115]	[0.121]	[0.146]	[0.133]	[0.140]	[0.169]
Trust People from State/Region							0.221+	-0.078	0.218							-0.009	0.073	0.290+
							[0.121]	[0.145]	[0.163]							[0.145]	[0.149]	[0.172]
Trust People from Country							-0.194+	-0.247+	-0.108							-0.008	-0.036	0.082
							[0.109]	[0.132]	[0.146]							[0.143]	[0.149]	[0.167]
Trust People from Other Country							0.134	0.391**	0.247+							0.08	0.207	0.133
							[0.100]	[0.122]	[0.135]							[0.131]	[0.137]	[0.162]
Trust Local Community							0.161	0.257+	0.075							0.005	0.023	-0.239
							[0.109]	[0.132]	[0.146]							[0.139]	[0.146]	[0.165]
PSYC Vulnerability							0.242	-0.001	-0.02							-0.238	-0.453+	-0.511
							[0.187]	[0.229]	[0.245]							[0.256]	[0.273]	[0.311]
Social Relationships Loss							0.033	0.074	0.05							0.647+	-0.164	0.172
							[0.257]	[0.308]	[0.339]							[0.383]	[0.404]	[0.454]
Employees/self-employed							0.013	-0.265	-0.172							0.309	0.14	0.225
							[0.183]	[0.223]	[0.247]							[0.211]	[0.221]	[0.251]
Professionals/Managers							-0.028	-0.207	-0.02							0.14	0.112	0.062
							[0.180]	[0.210]	[0.241]							[0.223]	[0.228]	[0.256]
Constant	-0.477	0.056	-0.704	-1.455**	-0.658	-1.589*	-2.139***	-1.477*	-2.282**	-0.165	0.083	1.988**	-0.798	-0.449	0.448	-1.135	-1.16	0.285
	[0.414]	[0.484]	[0.539]	[0.486]	[0.568]	[0.638]	[0.548]	[0.649]	[0.728]	[0.560]	[0.599]	[0.652]	[0.704]	[0.752]	[0.840]	[0.763]	[0.825]	[0.928]
LR chi2		95.58			155.33			187.33			145.93			202.62			212.3	
Observations		932			932			912			723			723			686	

Supplementary Table 5c | Econometric analysis of Charity Choice. Estimates of marginal effects from multivariate Probit model are reported. Standard errors are in brackets. See notes to Supplementary Table 5a and Supplementary Table 1 for variables' definition. *** p<0.001, ** p<0.01, * p<0.05, + p<0.10.

	United States & Italy											
	Model 1			Model 2			Model 3			Model 4		
DEP VAR: *P*	State/Region	Country	World	State/Region	Country	World	State/Region	Country	World	State/Region	Country	World
	(1)	(2)	(3)	(4)	(5)	(6)	(7)	(8)	(9)	(10)	(11)	(12)
Age	0.017***	-0.008	0.01	0.016***	-0.008+	0.007	0.016***	-0.008	0.006	0.017***	-0.008	0.007
	[0.004]	[0.005]	[0.005]	[0.004]	[0.005]	[0.006]	[0.004]	[0.005]	[0.006]	[0.004]	[0.005]	[0.006]
Female	0.346***	0.302**	0.142	0.356***	0.271*	0.106	0.366***	0.276*	0.121	0.349***	0.299**	0.144
	[0.097]	[0.109]	[0.120]	[0.099]	[0.111]	[0.123]	[0.099]	[0.112]	[0.124]	[0.102]	[0.114]	[0.126]
Education Level	-0.011	-0.051	0.013	-0.003	-0.062	0.007	-0.004	-0.068	0.003	-0.019	-0.058	0.001
	[0.042]	[0.048]	[0.052]	[0.043]	[0.048]	[0.053]	[0.043]	[0.048]	[0.053]	[0.047]	[0.053]	[0.058]
Small/Medium Metro Area	-0.021	-0.149	0.012	-0.022	-0.174	0.04	-0.021	-0.185	0.024	-0.009	-0.173	0.057
	[0.116]	[0.129]	[0.143]	[0.118]	[0.131]	[0.146]	[0.118]	[0.131]	[0.148]	[0.120]	[0.134]	[0.150]
Large Metropolitan Area	-0.151	0.03	-0.249	-0.187	-0.04	-0.235	-0.188	-0.06	-0.236	-0.147	-0.032	-0.177
	[0.149]	[0.162]	[0.185]	[0.151]	[0.165]	[0.189]	[0.152]	[0.166]	[0.192]	[0.156]	[0.171]	[0.195]
Rooted	-0.080	0.178	-0.379**	-0.083	0.137	-0.400**	-0.093	0.128	-0.418**	-0.097	0.043	-0.455**
	[0.124]	[0.146]	[0.147]	[0.126]	[0.147]	[0.150]	[0.126]	[0.148]	[0.151]	[0.130]	[0.151]	[0.154]
Conservative scale	-0.155***	-0.251***	-0.536***	-0.170***	-0.284***	-0.473***	-0.172**	-0.244**	-0.271**	-0.161**	-0.256***	-0.467***
	[0.046]	[0.053]	[0.063]	[0.050]	[0.058]	[0.068]	[0.062]	[0.076]	[0.085]	[0.052]	[0.061]	[0.070]
Income	0.047+	0.055*	0.009	0.032	0.056*	0.02	0.031	0.054+	0.023	0.018	0.048+	0.012
	[0.025]	[0.027]	[0.030]	[0.025]	[0.028]	[0.031]	[0.025]	[0.028]	[0.031]	[0.026]	[0.029]	[0.032]
Income Lost	-0.032	0.135	0.031	-0.037	0.106	0.011	-0.041	0.104	0.022	-0.066	0.095	-0.003
	[0.101]	[0.114]	[0.125]	[0.103]	[0.115]	[0.128]	[0.103]	[0.116]	[0.128]	[0.106]	[0.119]	[0.131]
Priming State/Region	0.132	-0.011	-0.043	0.168	-0.027	-0.023	0.165	-0.036	-0.026	0.242+	0.002	-0.008
	[0.138]	[0.153]	[0.174]	[0.140]	[0.155]	[0.179]	[0.140]	[0.156]	[0.181]	[0.144]	[0.160]	[0.184]
Priming Country	-0.109	-0.148	-0.09	-0.086	-0.151	-0.063	-0.088	-0.149	-0.08	-0.057	-0.161	-0.042
	[0.137]	[0.152]	[0.171]	[0.139]	[0.154]	[0.174]	[0.139]	[0.154]	[0.177]	[0.142]	[0.158]	[0.179]
Priming World	0.046	-0.172	0.317+	0.061	-0.18	0.360*	0.06	-0.186	0.364*	0.141	-0.138	0.434*
	[0.135]	[0.153]	[0.164]	[0.137]	[0.155]	[0.167]	[0.137]	[0.155]	[0.169]	[0.140]	[0.159]	[0.171]
County-level COVID Exposure	0.005	0.008	0.014	0.007	0.008	0.015+	0.007	0.007	0.016+	0.004	0.007	0.015
	[0.007]	[0.008]	[0.009]	[0.007]	[0.008]	[0.009]	[0.007]	[0.008]	[0.010]	[0.007]	[0.008]	[0.009]
Personal COVID Exposure	0.269**	0.11	0.350**	0.207*	0.088	0.274*	0.209	0.118	0.187	0.201+	0.062	0.264*
	[0.101]	[0.112]	[0.122]	[0.103]	[0.115]	[0.126]	[0.135]	[0.161]	[0.175]	[0.106]	[0.119]	[0.131]
Italy	0.303*	0.825***	0.827***	0.171	0.608***	0.529**	0.861	1.338*	1.731*	0.227	0.587***	0.556**
	[0.120]	[0.132]	[0.147]	[0.135]	[0.146]	[0.165]	[0.602]	[0.654]	[0.747]	[0.147]	[0.161]	[0.182]
Local Social Identity				0.478***	-0.203*	-0.125	0.502***	-0.086	-0.079	0.361***	-0.253*	-0.222*
				[0.074]	[0.086]	[0.095]	[0.093]	[0.114]	[0.125]	[0.085]	[0.099]	[0.109]
National Social Identity				-0.09	0.341***	-0.052	-0.04	0.228+	-0.004	-0.053	0.438***	-0.052
				[0.078]	[0.089]	[0.098]	[0.100]	[0.123]	[0.135]	[0.088]	[0.102]	[0.112]

(Continued)						United States & Italy						
	(1)	(2)	(3)	(4)	(5)	(6)	(7)	(8)	(9)	(10)	(11)	(12)
Global Social Identity				0.016	0.151*	0.501***	0.031	0.215*	0.406***	-0.046	0.074	0.425***
				[0.069]	[0.076]	[0.086]	[0.086]	[0.100]	[0.109]	[0.076]	[0.084]	[0.094]
Trust People from State/Region										0.124	0.009	0.250*
										[0.090]	[0.100]	[0.114]
Trust People from Country										-0.123	-0.139	-0.024
										[0.084]	[0.094]	[0.105]
Trust People from Other Country										0.117	0.302***	0.211*
										[0.078]	[0.088]	[0.099]
Trust Local Community										0.099	0.099	-0.097
										[0.084]	[0.094]	[0.105]
PSYC Vulnerability										0.067	-0.092	-0.248
										[0.147]	[0.168]	[0.185]
Social Relationships Loss										0.251	-0.049	0.123
										[0.208]	[0.237]	[0.264]
Employees/self-employed										0.111	-0.073	-0.042
										[0.135]	[0.151]	[0.169]
Professionals/Managers										0.071	-0.055	-0.008
										[0.135]	[0.148]	[0.167]
Conservative X Ita							-0.035	-0.109	-0.592***			
							[0.108]	[0.121]	[0.145]			
Personal Exposure X Ita							0.001	-0.046	0.179			
							[0.208]	[0.230]	[0.256]			
Local Social Identity X Ita							-0.05	-0.262	-0.044			
							[0.158]	[0.176]	[0.198]			
National Social Identity X Ita							-0.121	0.229	-0.24			
							[0.164]	[0.184]	[0.204]			
Global Social Identity X Ita							-0.048	-0.154	0.265			
							[0.142]	[0.155]	[0.181]			
Constant	-0.537+	-0.163	0.093	-1.363***	-0.691+	-0.926*	-1.559***	-0.899+	-1.333*	-1.862***	-1.310**	-1.435**
	[0.312]	[0.347]	[0.382]	[0.371]	[0.416]	[0.461]	[0.411]	[0.474]	[0.526]	[0.415]	[0.468]	[0.524]
LR chi2		266.84			381.31			416.21			398.31	
Observations		1,655			1,655			1,655			1,598	

Supplementary Table 5d | Econometric analysis of Charity Choice. Joint U.S. and Italy. Estimates of marginal effects from multivariate Probit model are reported. See notes to Supplementary Table 5a and Supplementary Table 1 for variable definition. Standard errors are in brackets. *** p<0.001, ** p<0.01, * p<0.05, + p<0.10

	United States	Italy
Age quota	0.7894	0.9233
Female	0.0188*	0.9868
Income	0.7484	0.8935
Education	0.4474	0.4356
Rooted	0.4041	0.928
Personal COVID Exposure	0.2124	0.7185
Conservative	0.5893	0.3351

Supplementary Table 6. Equality of Distribution of Key Variables by Prompts. P-values for Kruskal–Wallis tests are reported. The Kruskall-Wallis tests for the null hypothesis that samples in each prompt condition are from the same population for each of the variables listed above. The test is two-tailed.

	United States			Italy		
	Model 1			Model 2		
DEP VAR: SOCIAL IDENTITY	State	Country	World	Region	Country	World
	(1)	(2)	(3)	(1)	(2)	(3)
Age	0.004+	0.007***	-0.002	0.009***	0.005+	-0.001
	[0.002]	[0.002]	[0.002]	[0.003]	[0.003]	[0.003]
Female	-0.014	-0.014	0.076	0.07	0.150**	0.149**
	[0.048]	[0.045]	[0.051]	[0.053]	[0.053]	[0.052]
Education Level	0.016	-0.017	0.01	-0.070**	0.003	-0.006
	[0.020]	[0.019]	[0.021]	[0.024]	[0.024]	[0.024]
Small/Medium Metro Area	0.07	0.136*	0.03	0.013	0.07	-0.032
	[0.064]	[0.060]	[0.067]	[0.058]	[0.058]	[0.057]
Large Metropolitan Area	0.172*	0.197**	0.07	0.025	0.209**	0.056
	[0.079]	[0.074]	[0.083]	[0.079]	[0.079]	[0.078]
Rooted	0.052	0.119*	0.077	-0.025	0.032	-0.097
	[0.057]	[0.053]	[0.059]	[0.083]	[0.083]	[0.082]
Midwest	-0.208**	-0.178**	-0.012			
	[0.069]	[0.064]	[0.072]			
South	-0.111	-0.056	0.058			
	[0.070]	[0.065]	[0.073]			
West	0.006	-0.113+	-0.058			
	[0.070]	[0.065]	[0.073]			
South Italy				0.071	0.099+	0.091
				[0.058]	[0.058]	[0.057]
Conservative scale	0.056**	0.253***	-0.132***	0.106***	0.087**	-0.174***
	[0.021]	[0.019]	[0.022]	[0.029]	[0.029]	[0.029]
Income	0.036**	0.029**	0.005	0.047***	0.030*	-0.012
	[0.012]	[0.011]	[0.013]	[0.014]	[0.014]	[0.014]
Income Lost	0.025	0.107*	0.07	-0.022	0.026	0.088
	[0.049]	[0.046]	[0.052]	[0.057]	[0.057]	[0.056]
Priming State	-0.001	0.06	-0.103	-0.141+	0.002	-0.029
	[0.069]	[0.064]	[0.072]	[0.073]	[0.073]	[0.072]
Priming Country	-0.03	0.082	-0.074	-0.053	-0.034	0.005
	[0.068]	[0.064]	[0.071]	[0.074]	[0.074]	[0.073]
Priming World	-0.022	0.066	-0.119+	-0.035	0.014	-0.016
	[0.067]	[0.062]	[0.070]	[0.073]	[0.073]	[0.072]
County-Level COVID Exposure	0.002	-0.005	-0.007	-0.002	0.006+	-0.002
	[0.004]	[0.004]	[0.005]	[0.003]	[0.003]	[0.003]
Personal COVID Exposure	0.169***	0.082+	0.214***	0.166**	0.108*	0.116*
	[0.051]	[0.048]	[0.053]	[0.054]	[0.054]	[0.053]
Constant	1.747***	1.291***	2.508***	1.961***	2.077***	3.447***
	[0.157]	[0.147]	[0.165]	[0.187]	[0.187]	[0.185]
LR chi2		389.27			169.22	
Observations		932			723	

Supplementary Table 7 | Econometric Analysis of Social Identity. We fit a multivariate Tobit model (see notes to Supplementary Table 5a) to the indexes of social identity at state/region, country and world levels. See Supplementary Table 1 for variable definition. Standard errors are reported in brackets. *** p<0.001, ** p<0.01, * p<0.05, + p<0.10.

	Model 1			Model 2			Model 3			Model 4			Model 5			Model 6		
DEP VAR: AD	Region	Country	World	Region	Country	World	Region	Country	World	Region	Country	World	Region	Country	World	Region	Country	World
	(1)	(2)	(3)	(4)	(5)	(6)	(7)	(8)	(9)	(10)	(11)	(12)	(16)	(17)	(18)	(19)	(20)	(21)
Age	0.009***	-0.012**	0.00	0.006*	-0.010**	0.001	0.009**	-0.012**	0.00	0.008**	-0.011**	0.005	0.009**	-0.011**	0.002	0.006*	-0.011***	0.002
	[0.003]	[0.004]	[0.006]	[0.003]	[0.003]	[0.005]	[0.003]	[0.004]	[0.006]	[0.003]	[0.004]	[0.006]	[0.003]	[0.004]	[0.006]	[0.002]	[0.003]	[0.005]
Female	0.097+	-0.01	-0.093	0.103+	-0.009	-0.088	0.095	0.035	-0.154	0.087	0.003	-0.106	0.138*	0.07	-0.096	0.067	0.005	-0.013
	[0.056]	[0.072]	[0.108]	[0.053]	[0.067]	[0.099]	[0.061]	[0.077]	[0.120]	[0.059]	[0.077]	[0.113]	[0.068]	[0.082]	[0.123]	[0.051]	[0.066]	[0.096]
Education Level	0.003	0.003	0.047	-0.002	0.024	0.019	0.015	0.007	0.022	0	0.015	0.016	-0.001	0.013	0.029	-0.002	0.007	0.042
	[0.025]	[0.034]	[0.047]	[0.024]	[0.031]	[0.043]	[0.028]	[0.036]	[0.053]	[0.027]	[0.036]	[0.049]	[0.031]	[0.038]	[0.054]	[0.023]	[0.031]	[0.041]
Small/Medium Metro Area	0.053	-0.035	0.083	0.083	-0.032	0.01	0.089	-0.069	0.053	0.099	-0.056	-0.003	0.008	-0.123	0.021	0.075	-0.025	-0.003
	[0.060]	[0.081]	[0.117]	[0.057]	[0.075]	[0.106]	[0.067]	[0.086]	[0.130]	[0.065]	[0.086]	[0.122]	[0.073]	[0.091]	[0.133]	[0.055]	[0.073]	[0.102]
Large Metropolitan Area	0.012	0.15	-0.172	-0.004	0.143	-0.164	-0.016	0.205+	-0.216	-0.062	0.213+	-0.138	-0.095	0.183	-0.212	0.005	0.107	-0.164
	[0.085]	[0.104]	[0.165]	[0.081]	[0.095]	[0.152]	[0.094]	[0.110]	[0.184]	[0.091]	[0.110]	[0.172]	[0.104]	[0.115]	[0.188]	[0.078]	[0.094]	[0.145]
Rooted	-0.078	0.452**	-0.242	-0.081	0.363**	-0.18	-0.059	0.450**	-0.354*	-0.012	0.482***	-0.401*	-0.030	0.471**	-0.398*	-0.018	0.329**	-0.236+
	[0.087]	[0.138]	[0.156]	[0.081]	[0.127]	[0.139]	[0.094]	[0.143]	[0.171]	[0.091]	[0.145]	[0.159]	[0.105]	[0.153]	[0.174]	[0.080]	[0.127]	[0.136]
South Italy	0.054	-0.084	-0.127	0.028	-0.073	-0.069	-0.075	-0.014	0.003	-0.05	0.007	-0.051	-0.032	0.049	-0.015	0.088	-0.113	-0.127
	[0.062]	[0.081]	[0.117]	[0.059]	[0.074]	[0.107]	[0.067]	[0.084]	[0.129]	[0.065]	[0.085]	[0.121]	[0.074]	[0.090]	[0.132]	[0.056]	[0.073]	[0.103]
Conservative scale	0.105***	-0.003	-0.366***	0.102***	-0.013	-0.336***	0.151***	0.025	-0.534***	0.142***	0.013	-0.473***	0.091*	-0.058	-0.639***	0.084**	-0.031	-0.288***
	[0.031]	[0.042]	[0.072]	[0.030]	[0.040]	[0.067]	[0.034]	[0.044]	[0.082]	[0.033]	[0.045]	[0.077]	[0.037]	[0.046]	[0.089]	[0.028]	[0.039]	[0.064]
Income	-0.015	0.048*	-0.049+	-0.011	0.037*	-0.042	-0.004	0.041*	-0.05	-0.001	0.037+	-0.038	0	0.048*	-0.038	-0.018	0.047**	-0.032
	[0.015]	[0.019]	[0.029]	[0.015]	[0.018]	[0.026]	[0.017]	[0.021]	[0.032]	[0.016]	[0.021]	[0.030]	[0.018]	[0.021]	[0.032]	[0.014]	[0.018]	[0.025]
Income Lost	0.02	0.081	-0.163	-0.051	0.116	-0.067	-0.005	0.099	-0.107	0.04	0.105	-0.142	0.016	0.144+	-0.045	0.01	0.104	-0.099
	[0.060]	[0.077]	[0.115]	[0.056]	[0.071]	[0.106]	[0.065]	[0.081]	[0.128]	[0.064]	[0.082]	[0.121]	[0.073]	[0.087]	[0.132]	[0.054]	[0.069]	[0.100]
Priming Region	0.083	-0.047	-0.09	0.115	-0.051	-0.084	0.112	-0.097	-0.077	0.13	-0.105	0.056	0.151	-0.036	-0.067	0.086	-0.033	0.022
	[0.078]	[0.097]	[0.155]	[0.074]	[0.089]	[0.142]	[0.085]	[0.104]	[0.173]	[0.083]	[0.105]	[0.163]	[0.095]	[0.110]	[0.178]	[0.071]	[0.088]	[0.137]
Priming Country	0.105	-0.10	0.066	0.157*	-0.147	0.004	0.114	-0.145	0.09	0.064	-0.12	0.175	0.127	-0.073	0.073	0.106	-0.138	0.099
	[0.080]	[0.100]	[0.153]	[0.077]	[0.093]	[0.142]	[0.088]	[0.108]	[0.172]	[0.086]	[0.108]	[0.163]	[0.096]	[0.112]	[0.175]	[0.073]	[0.091]	[0.138]
Priming World	0.124	-0.265*	0.195	0.146+	-0.249**	0.156	0.073	-0.297**	0.350*	0.090	-0.285*	0.323*	0.127	-0.251*	0.345*	0.163*	-0.226*	0.13
	[0.078]	[0.104]	[0.145]	[0.075]	[0.096]	[0.133]	[0.086]	[0.110]	[0.164]	[0.083]	[0.111]	[0.153]	[0.095]	[0.117]	[0.167]	[0.071]	[0.094]	[0.128]

(Continued)	(1)	(2)	(3)	(4)	(5)	(6)	(7)	(8)	(9)	(10)	(11)	(12)	(16)	(17)	(18)	(19)	(20)	(21)
County-level COVID Exposure	-0.005	0.002	0.01	-0.005	0.001	0.011	-0.005	0.00	0.011	-0.006	0.002	0.008	-0.003	0.005	0.013	-0.005	0.001	0.008
	[0.004]	[0.005]	[0.008]	[0.003]	[0.005]	[0.008]	[0.004]	[0.005]	[0.009]	[0.004]	[0.005]	[0.009]	[0.004]	[0.005]	[0.009]	[0.003]	[0.005]	[0.007]
Personal COVID Exposure	0.048	-0.074	0.113	0.032	-0.095	0.118	0.018	-0.121	0.18	0.03	-0.124	0.187	0.083	-0.101	0.195	0.061	-0.071	0.1
	[0.057]	[0.073]	[0.110]	[0.054]	[0.068]	[0.102]	[0.062]	[0.078]	[0.123]	[0.060]	[0.079]	[0.115]	[0.069]	[0.083]	[0.127]	[0.051]	[0.066]	[0.097]
Efficiency Region	0.469***	-0.271**	-0.752***													0.272***	-0.206*	-0.438***
	[0.067]	[0.088]	[0.135]													[0.070]	[0.091]	[0.131]
Efficiency National	-0.321**	0.550***	-0.835***													-0.152	0.204*	-0.555**
	[0.099]	[0.102]	[0.200]													[0.102]	[0.100]	[0.189]
Efficiency World	-0.470***	-0.416**	0.550***													-0.299*	-0.145	0.139
	[0.132]	[0.148]	[0.149]													[0.126]	[0.141]	[0.131]
Goal Effectiveness Region				0.399***	-0.305**	-0.680***										0.221**	-0.235*	-0.395*
				[0.074]	[0.100]	[0.151]										[0.084]	[0.111]	[0.159]
Goal Effectiveness National				-0.434***	0.523***	-0.421**										-0.378***	0.396***	-0.155
				[0.090]	[0.097]	[0.151]										[0.098]	[0.107]	[0.160]
Goal Effectiveness World				-0.440***	- 0.618***	0.786***										-0.249*	-0.579***	0.543***
				[0.102]	[0.138]	[0.138]										[0.105]	[0.141]	[0.139]
Most Helping Self Region							0.137	-0.079	-0.076							-0.08	0.078	0.214
							[0.101]	[0.127]	[0.194]							[0.088]	[0.113]	[0.156]
Most Helping Self National							-0.285*	0.280*	0.082							-0.106	0.109	0.149
							[0.121]	[0.142]	[0.217]							[0.106]	[0.126]	[0.177]
Most Helping Self World							-0.312	-0.41	0.551							-0.053	-0.305	0.094
							[0.348]	[0.439]	[0.443]							[0.306]	[0.417]	[0.338]
People Most in Need Region										0.726***						0.395***		
										[0.142]						[0.117]		
People Most in Need National										-0.033	0.192	-0.817*				0.057	0.104	-0.42
										[0.099]	[0.124]	[0.392]				[0.083]	[0.102]	[0.297]
People Most in Need World										-0.191**	-0.137+	0.747***				-0.124*	-0.034	0.433***
										[0.065]	[0.083]	[0.127]				[0.056]	[0.071]	[0.108]

(Continued)	(1)	(2)	(3)	(4)	(5)	(6)	(7)	(8)	(9)	(10)	(11)	(12)	(16)	(17)	(18)	(19)	(20)	(21)
Expectation of Contribution Region													0.052			-0.015		
													[0.068]			[0.051]		
Expectation of Contribution National														-0.082			-0.128+	
														[0.081]			[0.066]	
Expectation of Contribution World															0.021			0.1
															[0.292]			[0.227]
Constant	-0.877***	-0.169	1.075**	-0.603**	-0.153	0.699+	- 0.806***	-0.37	0.847+	-0.677**	-0.38	0.277	- 0.973***	-0.593+	0.732+	-0.601**	0.1	0.304
	[0.210]	[0.280]	[0.390]	[0.198]	[0.264]	[0.362]	[0.239]	[0.309]	[0.462]	[0.219]	[0.292]	[0.402]	[0.249]	[0.310]	[0.431]	[0.203]	[0.274]	[0.368]
LR chi2		490.13			678.96			228.51			291.99			182.87			802.56	
Observations		556			556			556			556			714			550	

Supplementary Table 8 | Econometric analysis of charity characteristics effect on Aggregate Donations (*AD*). Answers to the open-ended questions from donors regarding the reason for donation are used to create measures of charity characteristics. See also notes to Supplementary Table 5a and Supplementary Table 1 for description of variables.

Charity guessed as experimenter preference	**Matches** (P|C)	**Nonmatches** (P|notC)
State/Region (n = 132)	35/111 (31%)	97/383 (25%)
National (n = 220)	65/136 (48%)	155/358 (43%)
International (n = 142)	52/144 (36%)	90/350 (26%)

Supplementary Table 9 | Frequency of charity guessed as experimenter's preference. n is the number of participants who answered positively to the question that experimenter had a preference over which charity the participant should chose, for each of the three charities primed in the instructions (see Supplementary Note SN6: Section I.16: Question 70, 70a, 70b). P|C is the probability that participants stated that the charity preferred by the researcher was the one primed in the instructions (see Supplementary Note SN6: Section I.2). P|notC is the probability that participants stated that the charity preferred by the researcher was one not primed in the instructions. (1- P|C- P|notC) is the residual probability that the participant stated that the researcher had no preference.

	United States & Italy
	**Model 1**
DEP VAR: *P*	(1)
Age	-0.005
	[0.005]
Female	-0.277*
	[0.111]
Education Level	-0.026
	[0.047]
Small/Medium Metro Area	0.165
	[0.133]
Large Metropolitan Area	0.321+
	[0.165]
Rooted	-0.092
	[0.142]
Italy	-0.012
	[0.130]
Conservative scale	0.252***
	[0.052]
Income	0.007
	[0.027]
Income Lost	0.200+
	[0.114]
Priming State/Region	0.074
	[0.162]
Priming Country	0.347*
	[0.157]
Priming World	0.334*
	[0.155]
Constant	-1.486***
	[0.349]
LR chi2	46.72
Observations	1,654

Supplementary Table 10 | Determinants of perception of experimenter's preference. A logit model was fitted having a dichotomous dependent variable taking value of either 1 if the participant answered affirmatively to the question whether the researcher had a preference over which charity the participant should have chosen, or 0 otherwise (see Supplementary Note SN6: Section I.2 and Section I.16: Question 70, 70a, 70b). The covariates are described in Supplementary Table 1. Standard errors are in brackets. *** p<0.001, ** p<0.01, * p<0.05, + p<0.10

Supplementary Figures

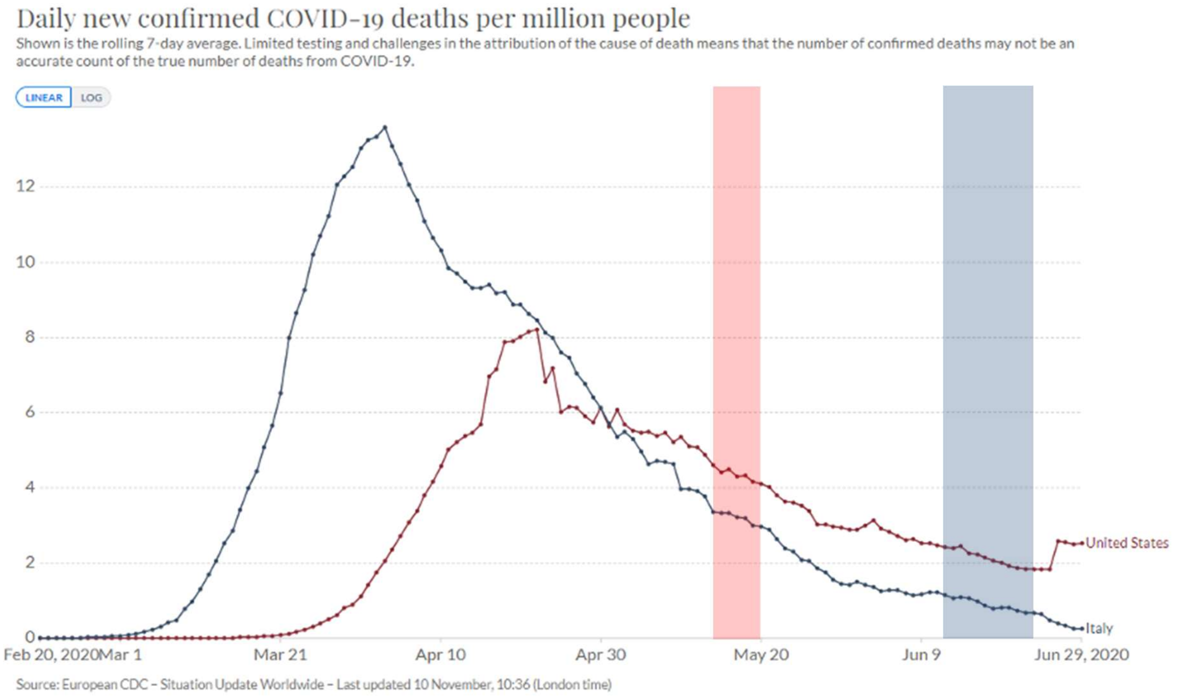

Supplementary Fig. 1: Evolution of death count per million people in the U.S. and Italy. The red and the blue lines plot the evolution of deaths per million people in the U.S. and Italy, respectively. The two bars denote the periods of data collection in the U.S. (red bar) and Italy (blue bar). Source: Ritchie et al., (2020): Our World in Data (https://ourworldindata.org/coronavirus/)[63], retrieved on 10th November 2020.

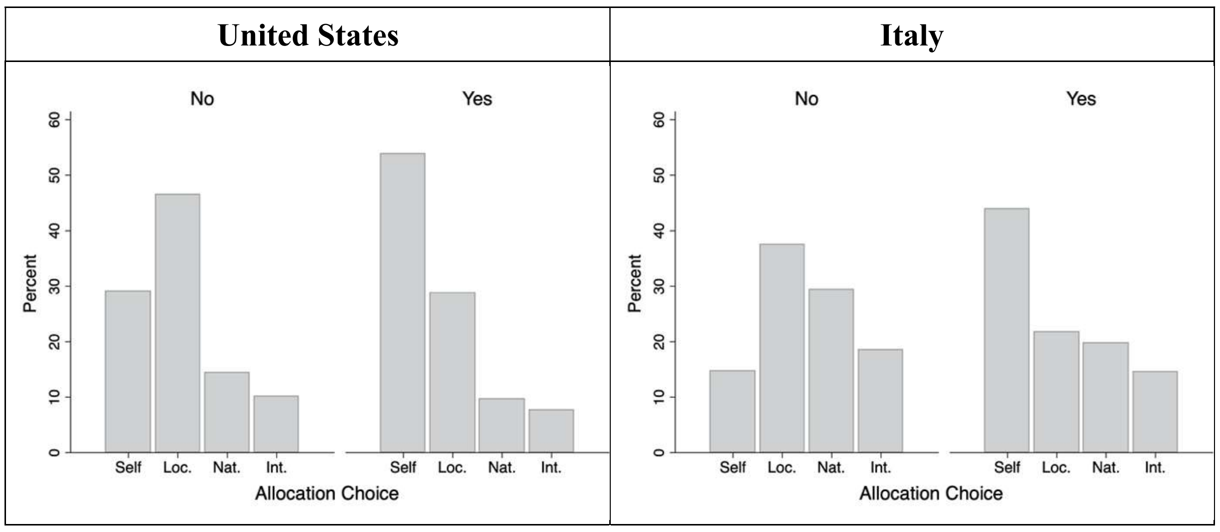

Supplementary Figure 2. Donation decisions as a function of perceived experimenter demand. The 'Yes' and 'No' panels of the figure refer to participants answering "Yes", or "No", respectively, to Question 70 of the survey: "*Do you think that the researchers preferred you to donate to one of the specific charities?*" (see Supplementary Note 5). The histograms plot the frequency of the allocation chosen by each group.

Supplementary Notes

SN1: Pre-registration of hypotheses

The original pre-registration of hypotheses and the pre-analysis plan are available at the Open Science Foundation repository at these links: https://osf.io/k74gm (for fieldwork in the U.S.) and https://osf.io/z82vc (for fieldwork in Italy). Since the Open Science Foundation did not allow the use of mathematical notation, nor the uploading of figures, we have deposited more reader-friendly versions of the pre-registration documents, which include mathematical notation, as well as a figure detailing the determination of the sample size based on power analysis, at this link: https://osf.io/jw46f/.

SN2: Analysis of effects of prompts on social identity

We conjectured that prompting the participant to frame the COVID-19 pandemic as being mainly relevant to one of the three levels considered (state/regional; or national; or international) would have increased social identity to the corresponding level, in comparison with baseline. Given the predicted positive effect of social identity on donations, we thus expected that prompting would have had an indirect positive effect on donations. We report the results of econometric analysis regarding this hypothesis in the Supplementary Table 7. The dependent variable was the social identity index (See Suppl. Table 1) and the set of covariates was the same as that used for other analyses. We found that our hypothesis was not supported, as priming was never significant at any level in comparison to baseline, in particular for the level of social identity that was primed. A plausible interpretation of this result is that the prompt manipulation did not override participants' prior perspective on the scope of the pandemic crisis. It is interesting to note that Personal COVID exposure had a significant effect in increasing social identity at all three levels in both countries.

SN3: Analysis of demographic effects and additional variables

Reading the results from Supplementary Table 2 in combination with those from Supplementary Table 5a permits the analysis of both participants' general propensity to donate and of how this is distributed across the three levels of donation.

Liberals were significantly more likely to donate in general, and particularly to world charities, than conservatives. This was the case in both countries, with the effect of conservatism being stronger in Italy than the U.S., after controlling for all other factors (Supplementary Table 2c, Supplementary Table 5b). This result supports other literature indicating that political leaning is a strong factor affecting pro-sociality and social attitudes[64]. While the effect of the Conservative scale was negative and highly significant for donations to the world charity in both the U.S. ($p=0.003$) and Italy ($p<0.001$), conservatives tended to give more than liberals to the regional charity in Italy ($p=0.021$), while they tended to give less, albeit insignificantly so, to the state-level charity in the U.S. ($p=0.12$). Conservative-leaning people also tended to give less to the national charity in the U.S. ($p=0.046$) and Italy, albeit insignificantly in the latter case ($p=0.27$) (Suppl. Table 5a, Model 1 for U.S. and Italy). The effect of the Conservative scale was significantly higher in Italy than the U.S. both for donations to the world charity ($p<0.001$), and for donations to the state/regional charity ($p=0.038$) (Suppl. Table 5b, Model 4). Overall, conservatives tended to keep all the bonus for themselves in both countries ($p<0.001$ for both countries), and to donate less once they made a donation ($p<0.001$ for both countries), in comparison to liberals (Suppl. Table 2a, Model 2 for U.S. and Italy).

Women were more generous than men in both countries. The tendency for females to donate more than men was only statistically significant at the local level in both the U.S. ($p=0.004$) and Italy ($p=0.032$) (Suppl. Table 5a, columns 1 and 10). Nevertheless, in the multivariate analysis coefficients for female donations to country and world charities were roughly similar in size and with the same sign (with the exception of world charity in Italy) to

the coefficient for the local charity, while standard errors were higher. This suggests that the lack of significance for female donations to country and world charity could be driven by coefficients being more imprecisely estimated, rather than as a weaker effect, as fewer participants chose the country and the world charity than state/regional charities.

When looking at donation amount there was an overall effect of age with older people giving more on average. However, the direction of the effect of age varied across the different charities (Supplementary Table 2a). In this case, the effect of Age was positive and highly significant for donations to state-level charity in both the U.S. ($p<0.001$) and Italy ($p=0.006$), while the effect of Age was negative and significant for donations to country-level charities in both the U.S. ($p=0.027$) and Italy ($p=0.014$). This means that older people tended to donate more to the state/regional level than younger ones, while the opposite occurred for donations to the national charities. The effect of Age for donations to world charities was instead indistinguishable from zero in both the U.S. ($p=0.88$) and Italy ($p=0.46$). It is remarkable that the coefficients of Age were the same in the two countries for each level (Suppl. Table 5a, Model 1 for U.S. and Italy).

As for other demographic and socio-economic variables, we found no effects for education and only sporadic effects for income in the U.S. but not in Italy, **with richer people tending to give more to the state level.** "Rooted" participants, that is, participants who were born in the country and whose parents were also born in the country, tended to give less to the world charity than "unrooted" participants, both in the U.S. ($p=0.018$) and Italy ($p=0.032$). While rooted Italians donated significantly more than "unrooted" Italians to the national charity ($p=0.001$), there was no such effect in the U.S. ($p=0.84$) (Suppl. Table 5a, Models 1 for U.S. and Italy). Initial runs of the model for the U.S. data included dummy variables for race, but these had no significant effects and were taken out of the models in order to maintain comparability with the Italian analyses. Having experienced income losses due to COVID-19

did not affect either propensity to donate or patterns of donations, as this variable was never significant (Suppl. Tables 2 and 5a). Likewise, no effect of the area of residence could be detected in either country.

In our pre-analysis plan we hypothesized that trust in people from the same state/region as the participant, country, or world, may have affected propensity to donate at the different levels. We report the results of this analysis in Supplementary Table 5a, Models 3, in a multivariate Tobit model including the same set of regressors as that used in previous analyses, also including social identity indexes. These variables were generally insignificant predictors of donation to any level, with a few exceptions (for instance, Trust in Local Community predicted donations to the state in the U.S., p=0.049). Some of these variables would have had higher significance had social identity indexes not been included as covariates, but they lost significance when controlling for social identity. Conversely, the coefficients of social identity variables remained nearly unaffected by the inclusion of additional covariates, as can be seen comparing Models 2 and 3 in Supplementary Table 5a. We also conjectured that an index of psychological vulnerability, and the experience of losses in social relationships (see Suppl. Table 1 for variables' definition), may have affected the patterns of donation. However, this did not prove to be the case after we controlled for all other factors. The participant's position in the labor market was also insignificant (Suppl. Table 5a, Models 3). Finally, we note that all the above results tended to be qualitatively similar when we analyzed the probability of choosing a particular charity, rather than the *AD* to that charity, in multivariate Probit models (Suppl. Tables 5c-d).

SN4: Analysis of charity characteristics

The results reported in the paper were based on Model 6 in Supplementary Table 8, in which all the variables pertaining to charity characteristics were simultaneously included. Results were qualitatively similar if each set of variables pertaining to each variable was considered in

isolation from all others, as done in the five other models of Supplementary Table 8. Not surprisingly, significance levels were generally higher in the latter case, as charity characteristics were positively correlated with each other.

SN5: Analysis of experimenter demand effects

We investigated the existence of possible experimental demand effects associated with the framing manipulation through responses to two questions asked at the very end of the questionnaire.

The first question asked whether the participant thought that the researcher had any preference about which charity the participant should have chosen. The second question was asked conditionally on the participant answering affirmatively to the first question, and asked the participant to state which charity the researcher would have preferred the participant to choose (See Supplementary Note SN5: Section I.16: Questionnaire Q70-70b).

In response to the first question, approximately 70% of respondents answered "No" in both the U.S. and Italy. Of those who responded "Yes" (n = 494), 103 were in the Control condition where no reference to any of the ingroups (state/region, nation, or world) had been given. For those in the three experimental conditions who said "Yes" (n = 391), we assessed whether their guess about the experimenters' preference corresponded to the frame they had received. Only 39% of guesses matched the level of ingroup that had been mentioned in the paragraph they read.

Supplementary Table 9 reports the proportion of respondents who guessed a charity that corresponded to the framing condition they had been in ("matches") compared to the proportion who named that charity among those in other conditions ("non-matches"). For

those who ventured a guess, the probability that the guess matched the frame was consistently higher than the proportion of mismatch guesses, but the difference was very small and statistically significant only for the international guesses [z(state)= 1.30, p = 0.19; z(nation)= 0.90, p = 0.37; z(world)= 2.32, p = 0.02]. In total, only 9% of our participants expressed a belief in experimenter demand and correctly named the charity that corresponded to the framing manipulation they had been exposed to.

Match and Mismatch Guesses

Although there is little evidence from the above analysis that many participants had accurately picked up a demand signal from the experimental manipulation, we did examine further whether their perception of experimenter intent influenced choices among the four options available (keep the money for self; donate to state/region charity; donate to national charity; donate to international charity).

First, we compared those who had answered "Yes" to the question about experimenter preference to those who said "No." As is evident in Supplementary Figure 2, the percentage of people deciding to keep the bonus for oneself was disproportionately higher in the former group. The null hypothesis of equality of distributions was rejected in both countries (Mann-Whitney-Wilcoxon (MWW) test: $p<0.001$, N=932 in the U.S., $p<0.001$, N=723 in Italy). The distribution among the group answering "Yes" was similar in the two countries and a Mann-Whitney test failed to reject the null of equality of distributions between countries (MWM: p=0.057; N=494). We fitted a logit model to explore which factors were most associated with the perception of a researcher preference (Yes vs No). Results are reported in Supplementary Table 10. Conservatives were significantly more likely to perceive a bias than liberals, and men more likely than women. Participants who were given the National and World frame were also significantly more likely to perceive a bias than those in the Control condition, but framing condition did not affect which charity they chose. Finally, we examined whether

there was a relationship between which charity the respondent named and the distribution of choices. For instance, did those who guessed that the researchers had a preference for the country charity, actually choose country with higher (or lower) frequency than others? For all three levels, we failed to reject the null hypothesis of equality of distribution between participants who had guessed one of the charities compared to those who had not guessed that charity (chi2 tests on pooled sample: p=0.78 for state/region level; p=0.91 for national level; p=0.21 for world level).

Overall, there was an association between perceiving an experimenter demand effect and choosing not to donate, and this reactance effect was particularly evident in the world frame condition. However, the number of respondents showing that pattern was very small and did not significantly affect the decision results in our study.

SN6: Instructions

The questionnaires (for one randomly assigned treatment condition) can be accessed at:
http://moore.az1.qualtrics.com/jfe/form/SV_a5edTFcE7GB9OzY (U.S. version)
https://moore.az1.qualtrics.com/jfe/form/SV_5nbz0p9mVFq62KF/ (Italian version)
The questionnaire reported below merges the different versions of the questionnaire administered in the different treatments in the U.S. and Italy (English translation for the Italian questionnaire) on samples of national residents.
In the questions below:
<name of country> means that either "the US" or "Italy" was used;
<state/region> means that "state" was used for the US, and "region" in Italy;
<name of previously selected state in the US / region in Italy> reported the state or region selected in Q12;
Text reported under titles ***<Control condition>, <Local condition>, <National condition>, <World condition>*** was administered to participants who had been randomly assigned to one of the four conditions, following a between-subject design. Qualtrics randomly assigned participants to a condition, following a fixed sequence whose order had been previously randomized.
Headlines in bold italics such as ***<Framing>*** were not read by participants, but mark the different sections of the questionnaire.- Notes in italics between the symbols < > were also not read by participants.

Life in the time of COVID19

<I.1. Initial Demographics>

Q1 Please enter your Prolific ID here

____

Q2 What is your sex?
- ○ Male
- ○ Female
- ○ Other

Q3 What year were you born?

____

Q4 Were you born in <Name of country>?
- ○ Yes
- ○ No

(If No in Q4:) Q4a Which country were you born in?

(If No in Q4:) Q4b In which year did you come to <Name of country>?

Q5 What is your <Name of country> citizenship status?
- ○ Citizen
- ○ Not a citizen
- ○ Rather not answer

Q6 Was your **mother** born in <Name of country>?
- ○ Yes
- ○ No

Q7 Was your **father** born in <Name of country>?
- ○ Yes
- ○ No

Q8 Have you ever resided outside <Name of country> for more than a year?
- ○ Yes
- ○ No

Q9 What is the highest level of education you completed?
- ○ Primary
- ○ High School/Secondary School
- ○ Some College
- ○ Technical School or Diploma, Trade Certificate or Other Post-high School Qualification other than University
- ○ Undergraduate Degree (e.g. BA, BS)
- ○ Masters
- ○ Doctoral Degree or Professional Degree

Q10 What is your marital status?

- Single
- Married
- Divorced/Separated
- Widowed
- Living with Partner

Q11 Do you live in a
- Large Metropolitan Area (More than 1.5 Million Inhabitants)?
- Medium-sized Metropolitan area (500,000 to 1.5 Million Inhabitants)?
- Small Metropolitan Area (200,000 to 500,000 Inhabitants)?
- Town (50,000 to 200,000 Inhabitants)?
- Village (Less than 50,000 Inhabitants)?
- Rural Area?

Q12 In which <state/region> do you currently reside?

Q13 What is your zip code?

____

<I.2 *Framing*>

The Corona virus (Covid-19) pandemic is clearly a major medical and economic crisis <*Local Condition*: **for <name of previously selected state in the US / region in Italy>>;** <*National Condition*: **for < the United States / Italy>>;** <*World Condition*: **for the world**>. In addition to the mounting death toll, this new virus has revealed how unprepared we are to cope with novel diseases that lead to serious illness and death for a large number of people all at the same time. **Medical facilities** <*Local Condition*: **around <name of previously selected state in the US / region in Italy>>;** <*National Condition*: **around <the United States / Italy>>;** <*World Condition*: **around the world**> **are being overburdened** with cases and medical staff are risking their own health to care for those who are critically ill. And the efforts we are making to slow the spread of infection have created **serious economic hardships** <*Local Condition*: **throughout <name of previously selected state in the United States / region in Italy>>;** <*National Condition*: **throughout <the United States / Italy>>;** <*World Condition*: **throughout the world**>. Loss of jobs and loss of business mean that families are in need and <Control Condition: we are>; <*Local Condition*: **<name of previously selected state in the US / region in Italy>> is;** <*National Condition*: **<the U.S. / Italy> is>;** <*World Condition*: **the world is**> facing economic recession. Coordinated efforts are needed to meet both the medical and economic crisis that this pandemic has created. **Cooperation** <*Local Condition*: **within <name of previously selected state in the US / region in Italy>>; within** <*National Condition*: **throughout <the United States / Italy>>;** <*World Condition*: **around the world**> **is essential** in this crisis because we are all in this together.

Q14a How serious do you believe the COVID-19 crisis to be <*Local Condition*: **for <name of previously selected state in the US / region in Italy>>;** <*National Condition*: **for <the United States / Italy>>;** <*World Condition*: **for the world**>?

- ○ Not At All Serious
- ○ Somewhat Serious
- ○ Moderately Serious
- ○ Very Serious
- ○ Extremely Serious

<I.3 Allocation choice>

Because this research is being conducted at the height of the corona virus pandemic crisis, the funders of the project have agreed to use part of the research funds to **provide opportunity for contributing to relief efforts**. As a participant in this study, you will be given a **bonus payment of <$5/€4>** in addition to the <$3/2.5€> payment that is the base pay for you completing this survey.
You may keep the bonus payment for yourself or **you can choose to donate some, all or none of it** to one of three charitable foundations that are providing food, medical and other assistance to individuals and families that have been seriously impacted by the pandemic.
The three options for donation are the following:· Charity A: money donated to this charity will go to an organization in **<name of previously selected state in the US / region in Italy>** to provide for those most affected by the pandemic across the state.· Charity B: money donated to this charity will go to a national organization to provide for those most affected by the pandemic across the United States.· Charity C: money donated to this charity will go to an international organization to provide for those most affected by the pandemic across the world.

If you choose to make a donation, you will first have to select one among those three options. Then you will be asked to indicate how much money you want to contribute toward that organization. This can be any amount up to $5.00. You will also have an option not to make any donation.
NOTE: For any amount of money you contribute, we will double that amount by a matching donation from our funds. So for instance:· If you contribute $3 of the $5 bonus payment to one of the three charities, we will add another $3 so that the total money donated to that charity will be $6 (and you keep $2 of the bonus payment for yourself); · If you contribute $1.50 to any of the charities, the charity will receive $3 total (and you keep $3.50 of the $5 bonus payment), and so forth.

<I.4: Comprehension check>

Q15-1 *<We only report values for USD in the following text. These values were converted into Euros in the Italian version of the questionnaire.>*

Suppose you select Charity B and donate $2. Below please indicate:
- How much money does Charity A receive?
- How much money does Charity B receive?
- How much money does Charity C receive?
- How much **bonus** money goes to you? (not including your $3 base pay) Remember for any amount of money you contribute, we will double that amount by a matching donation from our funds.

Charity A	▼ $0 ... $5
Charity B	▼ $0 ... $5
Charity C	▼ $0 ... $5
You	▼ $0 ... $5

<If answer is correct at first try:> Your answer is correct. Charity B will receive $4 and you will have $3 dollars as bonus. Charity A and Charity C will not get any donations.

<If answer is incorrect at first try:> **Your answer was mistaken.** Remember:
As a participant in this study, you will be given a bonus payment of $5 in addition to the $3 payment that is the base pay for you completing this survey.
You may keep the bonus payment for yourself or you can choose to donate some, all or none of it to one of three charitable foundations that are providing food, medical and other assistance to individuals and families that have been seriously impacted by the pandemic.
For any amount of money you contribute, we will double that amount by a matching donation from our funds.

Q15-2
<If answer is incorrect at first try:> Here is the test question again:
Suppose you select Charity B and donate $2. Below please indicate:
- How much money does Charity A receive?
- How much money does Charity B receive?
- How much money does Charity C receive?
- How much bonus money goes to you? (not including your $3 base pay)

Charity A	▼ $0 ... $5
Charity B	▼ $0 ... $5
Charity C	▼ $0 ... $5
You	▼ $0 ... $5

<*If answer is correct at second try:*> Your answer is correct. Charity B will receive $4 and you will have $3 dollars as bonus. Charity A and Charity C will not get any donations.

<*If answer is incorrect at second try:*> **Your answer was mistaken.** Remember:
- **only one charity** receives your donation; the others do not receive anything
- the chosen charity receives **double the amount you donated** (so if you donated $2, that charity receives $4)
- the amount you keep is **$5 minus the amount you donated**

Q15-3
<*If answer is correct at second try:*> Here is the test question again (If you do not get it correct this time unfortunately your participation in the survey will be discontinued and you will not receive any payment. Suppose you select Charity B and donate $2. Below please indicate:
- How much money does Charity A receive?
- How much money does Charity B receive?
- How much money does Charity C receive?
- How much bonus money goes to you? (<u>not</u> including your $3 base pay)

Charity A	▼ $0 ... $5
Charity B	▼ $0 ... $5
Charity C	▼ $0 ... $5
You	▼ $0 ... $5

<*If answer is correct at third try:*> Your answer is correct. Charity B will receive $4 and you will have $3 dollars as bonus. Charity A and Charity C will not get any donations.

<*If answer is incorrect at third try:*> Your response was incorrect again. Charity B would receive $4 and you would have $3 dollars as bonus. Charity A and Charity C would not get any donations.
We are sorry, your survey will be terminated and you will not receive any payment. Thank you.

<I.5: Decision>

NOW WE WANT YOUR ACTUAL DECISION...

Q16 To which organization do you want to contribute?

- ○ <name of previously selected state in the U.S. / region in Italy> aid organization
- ○ National aid organization
- ○ International aid organization
- ○ I do not want to donate

<If first option is selected:> Q17a How much money do you want to contribute to the <name of previously selected state in the U.S. / region in Italy> aid organization? (At the end of the survey you will be given our email address. If you want to receive certification of our donations to organizations, please write to us and we will be happy to show such certification when our survey is completed.)

Fill in any amount up to <$5.00 / €4.00> in <dollars/euros> and cents.

____

<If second option is selected:> Q17b How much money do you want to contribute to this national aid organization? (At the end of the survey you will be given our email address. If you want to receive certification of our donations to organizations, please write to us and we will be happy to show such certification when our survey is completed.)

Fill in any amount up <$5.00 / €4.00> in <dollars/euros>and cents.

____

<If third option is selected:> Q17c How much money do you want to contribute to this global aid organization? (At the end of the survey you will be given our email address. If you want to receive certification of our donations to organizations, please write to us and we will be happy to show such certification when our survey is completed.)

Fill in any amount up to <$5.00 / €4.00> in <dollars/euros>dollars and cents.

____

Thank you. Now we will go on with the remainder of the survey.

<I.6: Exposure to COVID19>

Q18a Have you been diagnosed with COVID-19?
- ○ Yes
- ○ No

Q18b Have you been hospitalized?
- ○ Yes
- ○ No

Q19a Has someone you live with been diagnosed with COVID-19?
- ○ Yes
- ○ No
- ○ Do not know

Q19b Have they been hospitalized?
- ○ Yes
- ○ No
- ○ Do not know

Q20a Has a family member or a close friend (not living with you) been diagnosed with COVID-19?
- ○ Yes
- ○ No
- ○ Do not know

Q20b Have they been hospitalized?
- ○ Yes
- ○ No
- ○ Do not know

Q21a Has any of your neighbors, acquaintances or colleagues diagnosed with COVID-19?
- ○ Yes
- ○ No
- ○ Do not know

Q21b Have they been hospitalized?
- ○ Yes
- ○ No
- ○ Do not know

Q22 Do you have a family member, neighbor, acquaintance or colleague who died from COVID-19?
- ○ Yes
- ○ No
- ○ Do not know

Q23 Please pick the option that best describes you
I worry about getting infected with Covid-19

- ○ Always
- ○ Most of the time
- ○ About half the time
- ○ Sometimes
- ○ Never

Q24 I feel vulnerable to Covid-19 infection

- ○ Strongly agree
- ○ Somewhat agree
- ○ Neither agree nor disagree
- ○ Somewhat disagree
- ○ Strongly disagree

Q25 I worry about my local community being infected with Covid-19

- ○ Always
- ○ Most of the time
- ○ About half the time
- ○ Sometimes
- ○ Never

<I.7: Connectedness>

Q26a **Prior** to the COVID-19 pandemic, how often did you socialize with friends **in person**?
- ○ Daily
- ○ Several days a week
- ○ Once a week
- ○ Less than once a week
- ○ Never

Q26b **Since** the COVID-19 pandemic, how often do you socialize with friends **in person** (compared to before)?
- ○ Much more
- ○ Somewhat more
- ○ About the same
- ○ Somewhat less
- ○ Much less

Q27a **Prior** to the COVID-19 pandemic, how often did you use the **phone, text or internet** to connect with friends who live in **<name of previously selected state in the U.S. / region in Italy>**?
- ○ Daily
- ○ Several days a week
- ○ Once a week
- ○ Less than once a week
- ○ Never

Q27b **Since** the COVID-19 pandemic, how often do you use the **phone, text or internet** to connect with friends who live in **<name of previously selected state in the U.S. / region in Italy>** (compared to before)?
- ○ Much more
- ○ Somewhat more
- ○ About the same
- ○ Somewhat less
- ○ Much less

Q28a **Prior** to the COVID-19 pandemic, how often did you use the **phone, text or internet** to connect with friends who live **<in other states in the U.S.> or <other regions in Italy>**?
- ○ Daily
- ○ Several days a week
- ○ Once a week
- ○ Less than once a week
- ○ Never

Q28b **Since** the COVID-19 pandemic, how often do you use the **phone, text or internet** to connect with friends who live in **<other states in the U.S.> or <other regions in Italy>** (compared to before)?

- ○ Much more
- ○ Somewhat more
- ○ About the same
- ○ Somewhat less
- ○ Much less

Q29a **Prior** to the COVID-19 pandemic, how often did you use the **phone, text or internet** to connect with friends who live **in other parts of the world**?

- ○ Daily
- ○ Several days a week
- ○ Once a week
- ○ Less than once a week
- ○ Never

Q29b **Since** the COVID-19 pandemic, how often do you use the **phone, text or internet** to connect with friends who live in **other parts of the world** (compared to before)?

- ○ Much more
- ○ Somewhat more
- ○ About the same
- ○ Somewhat less
- ○ Much less

Q30 Is either your mother or your father or both residing outside the **<Name of country>**?

- ○ Yes
- ○ No

Q31 **In the last year, prior** to the COVID-19 pandemic, how often did you engage in **volunteer activities**?

- ○ Daily
- ○ A few times a week
- ○ A few times a month
- ○ A few times a year
- ○ Never

Q32 **Since** the COVID-19 pandemic, how often do you engage in **volunteer activities** (compared to before)?

- ○ Much more
- ○ Somewhat more
- ○ About the same
- ○ Somewhat less
- ○ Much less

Q33 How many organizations are you actively involved in outside of work? (This includes social, professional, athletic, religious, civic, political organizations, etc.).

- ○ 1-2
- ○ 3-4
- ○ 5-6
- ○ More than 6
- ○ None

Q34a **Prior** to the COVID-19 pandemic, in an average month, how often did you **attend meetings of these organized groups (outside of work-related meetings)**?

- ○ Daily
- ○ A few times a week
- ○ A few times a month
- ○ Once a month
- ○ A few times a year
- ○ Never

Q34b **Since** the COVID-19 pandemic, in an average month, how often do you **attend meetings of these organized groups (outside of work-related meetings compared to before)**?

- ○ Much more
- ○ Somewhat more
- ○ About the same
- ○ Somewhat less
- ○ Much less

Q35a **Prior** to the COVID-19 pandemic, how strongly did you feel **connected to people in your neighborhood**?

- ○ Not at all
- ○ Somewhat not connected
- ○ Neither connected nor not connected
- ○ Somewhat connected
- ○ Very connected

Q35b **Since** the COVID-19 pandemic, how strongly do you feel **connected to people in your neighborhood**?

- ○ Not at all
- ○ Somewhat not connected
- ○ Neither connected nor not connected
- ○ Somewhat connected
- ○ Very connected

Q36 Who lives with you in your household? Check all that apply:

- ○ I live alone
- ○ My partner or spouse
- ○ My Parents
- ○ One child

- Two children
- Three or more children
- A friend or relative
- Multiple friends or relatives

<I.8: Social Identity>

Q37a How strongly do you feel attachment to **<name of previously selected state in the U.S. / region in Italy>**?
- Not at all
- Somewhat
- Strongly
- Very strongly

Q37b How strongly do you define yourself as a member of the **<name of previously selected state in the U.S. / region in Italy>** community?
- Not at all
- Somewhat
- Strongly
- Very strongly

Q37c How close do you feel to other members of the **<name of previously selected state in the U.S. / region in Italy>** community?
- Not at all
- Somewhat
- Strongly
- Very strongly

Q38a How strongly do you feel attachment to the **<Name of country>**?
- Not at all
- Somewhat
- Strongly
- Very strongly

Q38b How strongly do you define yourself as a member of the **<Name of country>** community?
- Not at all
- Somewhat
- Strongly
- Very strongly

Q38c How close do you feel to other members of the **<Name of country>** community?
- Not at all
- Somewhat
- Strongly
- Very strongly

Q39a How strongly do you feel attachment to the **world as a whole**?
- Not at all
- Somewhat
- Strongly
- Very strongly

Q39b How strongly do you define yourself as a member of the **world** community?
- Not at all
- Somewhat
- Strongly
- Very strongly

Q39c How close do you feel to other members of the **world** community?
- Not at all
- Somewhat
- Strongly
- Very strongly

<I.9: General Trust>

Q40 How much do you trust people from your **local community** in general?

	1 - I don't trust them at all	2 (2)	3	4	5 - I completely trust them
1	○	○	○	○	○

Q41 How much do you trust people from **<name of previously selected state in the U.S. / region in Italy>** in general?

	1 - I don't trust them at all	2 (2)	3	4	5 - I completely trust them
1	○	○	○	○	○

Q42 How much do you trust people the **<Name of country>** in general?

	1 - I don't trust them at all	2 (2)	3	4	5 - I completely trust them
1	○	○	○	○	○

Q43 How much do you trust people from **other countries** in general?

	1 - I don't trust them at all	2 (2)	3	4	5 - I completely trust them
1	○	○	○	○	○

<I.10: Immigration and Environment>

Q44 Please express your view on the following scale. The closer you are to statement (a), check a value on the scale close to 1. The closer you are to statement (b), check a value on the scale close to 5.

	1	2	3	4	5	
(a) Immigrants today make our country stronger because of their work and talents.	○	○	○	○	○	(b) Immigrants today are a burden on our country because they take our jobs and social benefits.

Q45 How important is it to protect the environment to you personally

- ○ Very important
- ○ Quite important
- ○ Somewhat important
- ○ Not very important
- ○ Not at all important

Q46 Suppose you were given another <$5/4€> bonus by the researchers. Would you donate some or all of this to an environmental organization (if the researchers would match your donation so that the organization received twice as much)?

- ○ Yes
- ○ No

Q46a How much would you choose to donate to the environmental organization? Enter any amount up to <$5.00 / €4.00> in <dollars/euros> and cents.

____

Q47 How willing would you be to pay higher prices in order to protect the environment?

- ○ Very willing
- ○ Fairly willing
- ○ Neither willing nor unwilling
- ○ Fairly unwilling
- ○ Very unwilling

Q48 As restrictions are lifted from this pandemic, which of these statements comes closer to your own point of view? The closer you are to statement (a), check a value on the scale close to 1. The closer you are to statement (b), check a value on the scale close to 5.

	1	2	3	4	5	
(a) Protecting the environment should be given priority, even if it causes slower economic growth and some loss of jobs.	○	○	○	○	○	(b) Economic growth and creating jobs should be given priority, even if the environment suffers to some extent.

Q48bis During the past month have you cut down your water consumption (for example not leaving water running when washing the dishes or taking a shower, etc.) for environmental reasons?

- ○ Always
- ○ Most of the time
- ○ About half the time
- ○ Sometimes
- ○ Never

<I.11: Globalization>

Q49 The world becoming more connected through greater economic trade and business ties is

- Very good
- Good
- Neither good nor bad
- Bad
- Very bad

Q50 The world becoming more connected through faster communication and greater movements of people is

- Very good
- Good
- Neither good nor bad
- Bad
- Very bad

Q51 All things considered, how satisfied are you with your life as a whole these days?

- Extremely satisfied
- Somewhat satisfied
- Neither satisfied nor dissatisfied
- Somewhat dissatisfied
- Extremely dissatisfied

<I.12: Economic Vulnerability>

Q52 What is your current working situation (paid work other than working on these surveys)?

- Working at my workplace
- Working from home
- Not currently working

Q53 What is your usual employment situation?

- Full-time employed
- Part-time employed
- Self-employed
- Retired/Pensioned
- Housewife/husband not otherwise employed
- Student
- Unemployed
- On disability
- Other

Q54 Have you lost income or gained income because of COVID-19, or has it stayed stable?
- Lost
- Gained
- Stable

Q55 What percentage of your income have you lost?
- Less than 10%
- 10% to less than 20%
- 20% to less than 40%
- 40% to 60%
- More than 60%

Q56 What percentage of your income have you gained?
- Less than 10%
- 10% to less than 20%
- 20 to less than 40%%
- 40% to 60%
- More than 60%

Q57a **Prior** to the COVID-19 pandemic, when it came to the **financial situation of your household,** what were your expectations for the 12 months to come, would the next 12 months be better, worse, or the same?
- Worse
- The same
- Better

Q57b **Since** the COVID-19 pandemic, when it comes to the **financial situation of your household,** what are your expectations for the 12 months to come, will the next 12 months be better, worse, or the same?
- Worse
- The same
- Better

Q58a **Prior to t**he COVID-19 pandemic, how likely did you believe it was that you would **still have a job** in 6 months (if you had a job then)?
- Very likely
- Likely
- Unlikely
- Very unlikely
- Not applicable - Did not have a job

Q58b **Since** the COVID-19 pandemic, how likely do you believe is it that you will **still have a job** in 6 months (if you have one now)?

- ○ Very likely
- ○ Likely
- ○ Unlikely
- ○ Very unlikely
- ○ Not applicable - Did not have a job

<I.13: Trust in Politicians>

Q59 Has your trust in politicians in **<name of previously selected state in the U.S. / region in Italy>** increased, decreased, or stayed stable for their handling of COVID-19?

- ○ Increased
- ○ Stayed the same
- ○ Decreased

Q60 Has your trust in politicians **nationally** increased, decreased, or stayed stable for their handling of COVID-19?

- ○ Increased
- ○ Stayed the same
- ○ Decreased

Q61 Has your trust in politicians **globally** increased, decreased, or stayed stable for their handling of COVID-19?

- ○ Increased
- ○ Stayed the same
- ○ Decreased

Q62 During this COVID-19 pandemic, have you received food, money, or other assistance from any of the following:

	Check all that apply
Friends or neighbors	○
A charity (such as a food bank)	○
State government	○
Federal government	○
None	○

<I.14: Political Views>

Q63 To what extent do you agree with the following statement? “People like me don’t have any say about what the government does”

- ○ Strongly disagree
- ○ Disagree
- ○ Neither agree nor disagree
- ○ Agree
- ○ Strongly agree

Q64 In political matters, people often talk of “Liberal” and “Conservative.” Generally speaking, how would you place your views on this scale?

- ○ Very Liberal
- ○ Moderately liberal
- ○ Neither liberal nor conservative
- ○ Moderately conservative
- ○ Very conservative

Q65 Generally speaking, do you think of yourself as a Democrat, Independent, Republican, or something else?

- ○ Democrat
- ○ Independent
- ○ Republican
- ○ Something else
- ○ No party

Q65bis Generally speaking, how would you define your political orientation?

- ○ Lega
- ○ PD
- ○ Movimento 5 Stelle
- ○ Fratelli d'Italia
- ○ Forza Italia
- ○ Something else (specify at next question)
- ○ No party

(If Something else is selected:) Please indicate your political orientation:

____

<I.15: Demographics>

<*Question only asked in the U.S.:*> Q66 How would you define your ethnicity? (Choose all that apply)

- ○ White
- ○ African American
- ○ White Hispanic
- ○ Other Hispanic
- ○ Asian or Asian American
- ○ Native American
- ○ Middle Eastern
- ○ Other (Please specify at next question)

<*Question only asked in the U.S.:*> Q66a Ethnicity if you chose other:

____

Q66bis1 In which country was your **mother** born?

Q66bis2 In which country was your **father** born?

Q67 Look at the following categories. What is the category that best represents your current or last occupation? (Choose only one answer)

- ○ Managers
- ○ Professionals
- ○ Technicians and Associate Professionals
- ○ Clerical Support Workers
- ○ Service and Sale Workers
- ○ Skilled Agricultural, Forestry and Fishery Workers
- ○ Craft and Related Trades Workers
- ○ Plant and Machines Operators, and Assemblers
- ○ Unskilled Labor
- ○ Armed Forces Occupations

Q68 Here is a scale of incomes. We would like to know in what group your household is, counting all wages, salaries, pensions and other incomes that come in. Just check the group your household fell into in 2019, in terms of gross income before deductions.

- ○ $0 - $9,999
- ○ $10,000 - $14,999
- ○ $15,000 - $24,999
- ○ $25,000 - $34,999
- ○ $35,000 - $49,999
- ○ $50,000 - $74,999
- ○ $75,000 - $99,999
- ○ $100,000 - $149,999
- ○ $150,000 - $199,999
- ○ Over $200,000

Q68a Here is a scale of incomes. We would like to know in what group your household is, counting all wages, salaries, pensions and other incomes that come in. Just check the group your household fell into in 2019, in terms of gross income before deductions.

- ○ €0 - €5,999
- ○ €6.000 - €11,999
- ○ €12.000 - €23,999
- ○ €24.000 - €35,999
- ○ €36.000 - €47,999
- ○ €48.000 - €59,999
- ○ €60.000 - €71,999
- ○ €72.000 - €83,999
- ○ €84.000 - €99,999
- ○ Over €100.000

<I.15: Choice of Charity>*<Questions Q68b-j were only asked in Italy.>*

You have previously made a choice regarding the destination of your bonus to a charity active in your **region** | in **Italy** | in the **world**.

Q68b In your opinion, which type of charity is the most efficient, i.e. the one most capable of eliminating waste in the management of donations?

- ○ Regional aid organization
- ○ National aid organization
- ○ International aid organization
- ○ There are no differences

Q68c In your opinion, what type of charity is best able to achieve the goal of helping people affected by COVID-19?

- ○ Regional aid organization
- ○ National aid organization
- ○ International aid organization
- ○ There are no differences

Q68d In your opinion, what kind of charity is best able to help you and your family if you need it?

- ○ Regional aid organization
- ○ National aid organization
- ○ International aid organization
- ○ There are no differences

Q68e In your opinion, which people are most in need of help because of the COVID-19 epidemic?

- ○ People in the <name of previously selected region>
- ○ People in Italy
- ○ People from all over the world
- ○ There are no differences

Q68f Think about your choice of the charity to which you want to contribute, you had chosen to **donate to the <name of previously selected region>**. Imagine making this decision two months ago, during the peak of the Covid-19 outbreak. Would you have made the same choice again?

- ○ Yes
- ○ No

Q68g What would have been your choice then

- ○ <name of previously selected region> aid organization
- ○ National aid organization
- ○ International aid organization
- ○ I would not have donated

Q68h Think back to your choice of the charity to which to allocate your contribution, you had chosen **not to donate**. Imagine making this decision two months ago, during the peak of the Covid-19 epidemic. Would you have made the same choice again?

- ○ Yes
- ○ No

Q68i What would have been your choice then

- ○ <name of previously selected region> aid organization
- ○ National aid organization
- ○ International aid organization

Q68j How many euros would you have liked to contribute?

Fill in any amount up to €4.00 in euros and cents.

____

<I.16: Charitable Motivation>

Q69 Could you briefly explain what motivated your COVID-19 aid organization donation decision earlier in the survey?

____

Q70 Do you think that the researchers preferred you to donate to one of the specific charities?
- ○ Yes
- ○ No

Q70a Which one?
- ○ <name of previously selected state in the U.S. / region in Italy> Charity
- ○ National Charity
- ○ World Charity

Q70b Which charity do you think is the most chosen by other participants in this study as the recipient of donations?
- ○ Regional Charity
- ○ National Charity
- ○ World Charity

<I.17: Survey Modality>

Q80 Did you take the survey on a phone?
- ○ Yes
- ○ No

<I.18: Social Identity - Region Italy>

Q80bis Which region do you feel you belong to the most?

Qend Please contact us at studycontact@moore.sc.edu if you want to receive certification of our donations to organizations. We will be happy to show such certification when our survey is completed.

References

63. Ritchie, H., Ortiz-Ospina, E., Beltekian, D., Mathieu, E., Hasell, J., Macdonald, B., Giattino, C., Appel, C., Rodés-Guirao,L. & Roser, M. (2020) - "Coronavirus Pandemic (COVID-19)". Published online at OurWorldInData.org. Retrieved from: 'https://ourworldindata.org/coronavirus' [Online Resource]

64. Alesina, A., Stantcheva, S. & Teso, E. Intergenerational mobility and preferences for redistribution. *Am. Econ. Rev.* **108**, (2018)
